# Rearrangements of 2.5 Kilobases of Noncoding DNA from the *Drosophila even-skipped* Locus Define Predictive Rules of Genomic *cis*-Regulatory Logic

**DOI:** 10.1371/journal.pgen.1003243

**Published:** 2013-02-28

**Authors:** Ah-Ram Kim, Carlos Martinez, John Ionides, Alexandre F. Ramos, Michael Z. Ludwig, Nobuo Ogawa, David H. Sharp, John Reinitz

**Affiliations:** 1 Department of Ecology and Evolution, Chicago Center for Systems Biology, University of Chicago, Chicago, Illinois, United States of America; 2 Department of Biochemistry and Cell Biology, Stony Brook University, Stony Brook, New York, United States of America; 3 Department of Biochemistry, University of Cambridge, Cambridge, United Kingdom; 4 Escola de Artes, Ciências e Humanidades, Universidade de São Paulo, São Paulo, Brazil; 5 Genomics Division, Lawrence Berkeley National Laboratory, Berkeley, California, United States of America; 6 Theoretical Division, Los Alamos National Laboratory, Los Alamos, New Mexico, United States of America; 7 Department of Statistics, Department of Molecular Genetics and Cell Biology, and Institute of Genomics and Systems Biology, University of Chicago, Chicago, Illinois, United States of America; University of California Berkeley, United States of America

## Abstract

Rearrangements of about 2.5 kilobases of regulatory DNA located 5′ of the transcription start site of the *Drosophila even-skipped* locus generate large-scale changes in the expression of *even-skipped* stripes 2, 3, and 7. The most radical effects are generated by juxtaposing the minimal stripe enhancers MSE2 and MSE3 for stripes 2 and 3 with and without small “spacer” segments less than 360 bp in length. We placed these fusion constructs in a targeted transformation site and obtained quantitative expression data for these transformants together with their controlling transcription factors at cellular resolution. These data demonstrated that the rearrangements can alter expression levels in stripe 2 and the 2–3 interstripe by a factor of more than 10. We reasoned that this behavior would place tight constraints on possible rules of genomic *cis*-regulatory logic. To find these constraints, we confronted our new expression data together with previously obtained data on other constructs with a computational model. The model contained representations of thermodynamic protein–DNA interactions including steric interference and cooperative binding, short-range repression, direct repression, activation, and coactivation. The model was highly constrained by the training data, which it described within the limits of experimental error. The model, so constrained, was able to correctly predict expression patterns driven by enhancers for other *Drosophila* genes; *even-skipped* enhancers not included in the training set; stripe 2, 3, and 7 enhancers from various Drosophilid and Sepsid species; and long segments of *even-skipped* regulatory DNA that contain multiple enhancers. The model further demonstrated that elevated expression driven by a fusion of MSE2 and MSE3 was a consequence of the recruitment of a portion of MSE3 to become a functional component of MSE2, demonstrating that *cis*-regulatory “elements” are not elementary objects.

## Author Summary

Metazoan genes, including those of humans, contain large noncoding regions that are required for viability. Sequence variations in these regions are statistically associated with human disease, but the mechanisms underlying these associations are not well understood. These regions regulate transcription and are frequently larger than the gene's transcript by an order of magnitude. In this paper we attempt to elucidate the regulatory code of these noncoding segments of DNA by means of quantitative spatially resolved gene expression data and a computational model. The expression data comes from the early embryo of the fruit fly *Drosophila melanogaster*. We chose a family of DNA constructs to analyze that drive very different patterns of expression when very small changes in DNA sequence are made, reasoning that this sensitivity would reveal important properties of the regulatory code. The model reproduced the training data with precision greater than the expected accuracy of the training data itself. It was able to correctly predict from DNA sequence the expression of 44 segments of DNA from many genes and species.

## Introduction

Understanding DNA encoding of the *cis*-regulatory logic responsible for controlling gene expression in metazoans is a problem at the heart of modern genomics. As yet, a precise and predictive decryption of this code comparable to the genetic code for protein structure has remained elusive. Nevertheless, it is known that the regulatory DNA which controls the transcription of genes in higher eukaryotes can frequently be divided into functionally distinct contiguous regions defined by their ability to direct expression independently when placed in reporter constructs. When assayed in this manner, each fragment directs gene expression in a particular tissue or spatio-temporal domain. The genomic regions corresponding to these DNA fragments are known as enhancers or *cis*-regulatory modules (CRMs). Enhancers are usually separated from one another by regions of DNA which cannot independently drive transcription. Enhancers typically contain clusters of binding sites for transcription factors (TFs). Enhancers can act over many kilobases (kb) from the transcription start site (TSS), and are still functional when orientation is reversed. Although some progress has been made in understanding the expression of individual enhancers, this understanding has not yet reached a level that is adequate for prediction. In particular, understanding individual enhancers is not sufficient, as it is now clear that multiple enhancers act simultaneously to ensure accurate and robust gene expression [1]–[3]. Indeed, a real solution of the *cis*-regulatory logic problem in metazoa requires understanding of the control of gene expression at the level of a whole, intact genetic locus. It is the whole locus and not the enhancer which is the fundamental unit of physiological function, and hence it is the whole locus and not the enhancer on which natural selection acts over evolutionary time.

What is missing from current efforts to gain an understanding of the control of transcription are the rules which determine whether, and to what extent, a particular configuration of bound factors will activate or repress transcription. These rules and the model based on them must be validated by comparison to quantitative data on TFs and their transcriptional outputs, and because single nuclei are the fundamental units of transcriptional processing, these data must be at nuclear resolution or from a group of cells in a uniform transcriptional state. In other words, to demonstrate an understanding of transcriptional control, it is necessary to be able to calculate the transcriptional response of a segment of DNA to an accuracy comparable to that observed *in vivo*. Such a calculation will involve both the DNA sequence and certain parameters determined by training on data. At the very minimum, given a set of DNA sequences and the expression patterns driven by them, one should be able to use the model to calculate the observed expression patterns with a residual error less than or equal to the likely error of the experimental observations themselves. A statistically significant correlation of the model output with expression data is an inadequate criterion of correctness—a highly correlated pattern is typically sufficiently different from wild type that it would cause death if expressed in a real organism. Beyond this minimal level, a more stringent test is the correct prediction of expression driven by segments of DNA not used for training. Finally, understanding will be demonstrated by performing these calculations of transcriptional output on DNA segments larger than classical enhancers, ideally on an entire locus.

In 2003 we began to address this question by proposing a model of transcriptional control which contains an explicit thermodynamic representation of the occupancies of individual binding sites as a function of the concentrations of the TFs [4]. We applied this model to the blastoderm of *Drosophila*, a syncytium in which transcriptional control operates at an extremely precise spatial level that approaches cellular resolution. By making use of previously obtained quantitative data on TF levels [5]–[7], we were able to satisfy not only the minimum criterion of calculating to within the margins of experimental error in measurements of quantitative gene expression, but also to extend our calculation beyond well-described enhancers to understand how expression of *Drosophila even-skipped* (*eve*) stripe 7 was driven by the sequences not present in its “classical” enhancer [8]. Since that time, other modeling studies have been made on certain enhancers with small numbers of binding sites [9]–[13]. At a larger scale, Segal and coworkers modeled a set of previously described enhancers in the *Drosophila* segmentation system using the TF dataset employed in [8] together with *E. coli lac*Z reporter gene expression obtained from the literature and digitized in a binary zero/one manner [14]. A more recent study on this dataset made use of the correlation between data and model output to compare the roles of different transcriptional control mechanisms [15]. In both of these cases the calculation of transcriptional output from known sequences with trainable parameters resulted in expression patterns containing large qualitative errors that would be expected to result in *in vivo* lethality.

In this paper we develop and validate methods that lead to an improved understanding of transcriptional control. We augmented our previously published model [4], [8], which represented sequence specific binding of TFs, steric competition between bound factors, activation, short-range repression (also called “quenching”), and direct repression, by including coactivation and cooperative binding of TFs to DNA. We then applied our model to certain genes expressed in the blastoderm of *Drosophila*. By assembling many multi-channel scanned confocal images of embryos in this embryonic stage, we are able to construct a dataset at cellular resolution in which the concentrations of TFs and the corresponding transcription rate for a given gene or reporter in each blastoderm nucleus are determined to within a relative error of less than 10% [6], [8], [16]. This enables us to treat the *Drosophila* blastoderm as an *in vivo* microarrray in which it is possible to perform many transcription assays in parallel. These assays were performed on genes in a native chromosomal context in cells with well defined concentrations of TFs that produce markedly different transcriptional outputs from relatively small changes in TF concentration, resulting in an assay system of sensitivity and reproducibility unmatched by any tissue culture system we are aware of. We then challenged this assay system with a family of seven carefully selected rearrangements of two early acting enhancers of the *Drosophila eve* locus. Each rearrangement drives a different expression pattern, and the most informative patterns were quantitatively compared by transforming all constructs to a common chromosomal site and quantitatively assaying reporter expression together with the levels of nine TFs.

We were able to train the model on the data so that our calculations of training set expression are equivalent to observations within experimental error. Given these model parameters, we show how the different expression patterns observed in the rearrangements can be understood in terms of the interplay of multiple mechanisms acting in concert. From the model obtained from the training set of expression data, which was driven by only 2.5 kb of noncoding DNA subjected to certain rearrangements, we are able to predict with high accuracy the expression patterns driven by a variety of segments of DNA totaling 51 kb. These include *eve* enhancers from 16 Drosophilid and 6 Sepsid species, as well as enhancers for other *melanogaster* pair-rule and gap genes. In addition, the model was able to correctly predict the expression driven by the entire 3′ or 5′ early acting *eve* promoter, indicating that the predictive capability of the model extends to large, contiguous regions of DNA that contain multiple enhancers.

## Results

The results of this study are presented in four sections. In Section 1, we discuss the experimental system used to obtain training data together with the results obtained. In Section 2 we present our theory of transcriptional control. Section 3 contains an analysis of how well the model accounts for the training data and of its ability to correctly predict expression patterns driven by DNA sequences not used in training. Finally, in Section 4 we show explicitly how multiple regulatory mechanisms acting in concert give rise to the patterns of expression seen in the training data. Although all of this material is necessary to fully understand our study, a reader who wished to assess the performance of the model without delving into mathematical details might skip Sections 2 and 4 and read only Sections 1 and 3.

### Quantitative gene expression data at single nucleus resolution

**Figure 1:**
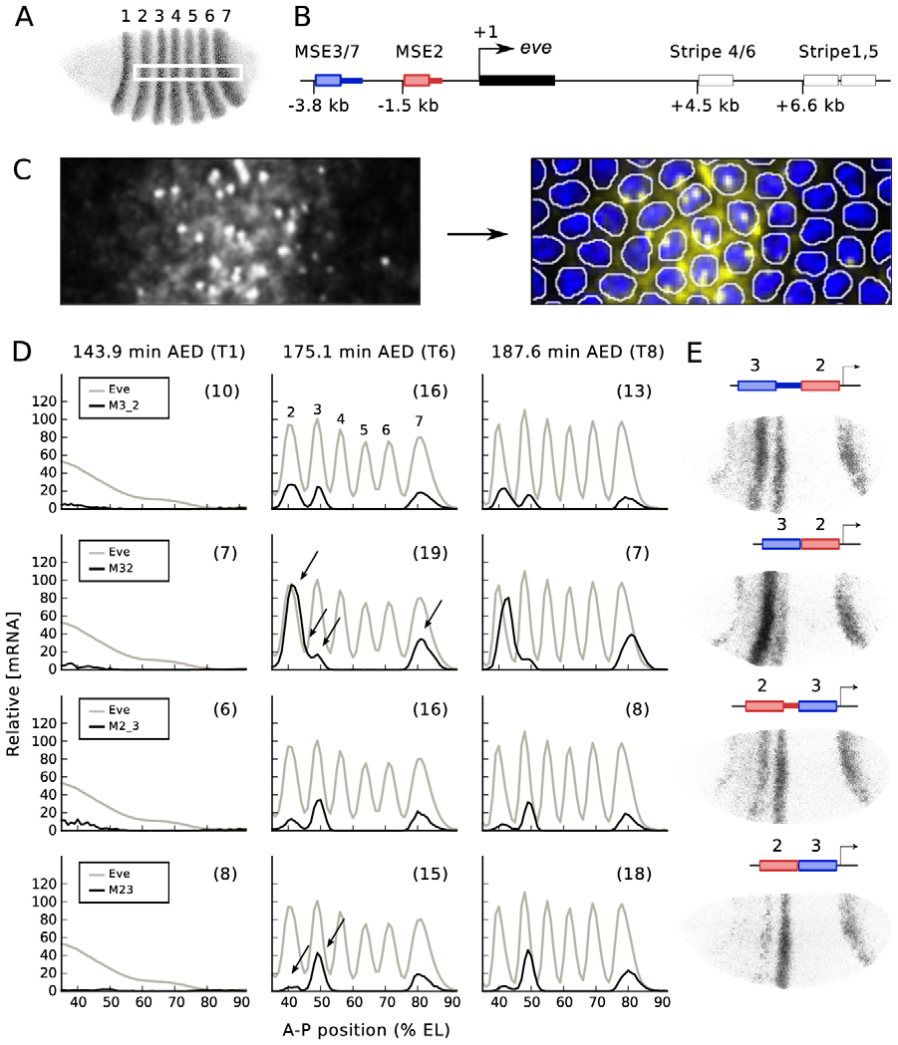
Fusion constructs. (A) The 7 striped expression pattern of *eve*, visualized with antibody staining. This and other embryos are oriented dorsal up and anterior to the left. The white rectangle located in the middle of the embryo indicates a 10% strip ranging from 35 to 92% embryo length (EL). (B) Schematic view of the *eve* gene. The transcript (black box) and early acting enhancers are shown. The distance of the 5′ end of each enhancer from the TSS is specified. The colored boxes and adjacent thick lines indicate the two segments of DNA used to create various reporter constructs. (C) (left) Fluorescence *in situ* hybridization for *lacZ* mRNA. (right) Segmented image with nuclear mask. Image segmentation was carried out as described [16]. Intense and punctate fluorescent spots in the nuclei are nascent transcripts. (D) Quantitative expression data for Eve protein and 4 fusion constructs, obtained from the area shown in the white rectangle in B. Embryos were classified temporally as belonging to one of eight time classes (T1–T8) in cleavage cycle 14A (C14A), each about 6.5 min long, as described [6]. T1, T6 and T8 data are shown here, with time after egg deposition (AED). The numbers in parentheses are the number of embryos used to generate the averaged expression profiles of each time class. Arrows indicate regions of major alteration in gene expression after spacer removal. (E) *lac*Z mRNA expression from individual embryos. 4 fusion constructs and their gene expression at T6 are shown.

We sought a small collection of regulatory DNAs which, by driving reporter expression of *lac*Z RNA, would provide the maximum amount of information on the rules of transcriptional control. *eve* is a logical source for such regulatory DNA because it is known that the 7 narrow stripes of gene expression (Figure 1A), each about 3 nuclei wide, form by the repressive action of gap gene encoded TFs such as Hunchback (Hb), Kruppel (Kr), Knirps (Kni) and Giant (Gt), expressed in domains 10–15 nuclei wide [6]. *eve* stripes 2 and 3 are particularly informative. It has been shown that stripe 2 is repressed by Kr, but stripe 3 evades repression by peak levels of Kr [17]. Hb, on the other hand, represses stripe 3 while it activates stripe 2 expression [18], [19]. These observations provide stringent mechanistic constraints on transcriptional regulation which can be made even more stringent by considering fusions of minimal enhancers expressing the two stripes.

Minimal stripe elements for stripes 2 and 3 (MSE2 and MSE3) can drive normal expression of both stripes if separated by as little as 155 bp (172 bp with polylinker) or 335 bp (360 bp with polylinker) of endogenous DNA 3′ of MSE2 or MSE3 respectively, but drive abnormal expression if these DNA fragments are removed [20]. While commonly referred to as “spacers”, these two segments of DNA are in no sense nonfunctional. We used the previously described reporter constructs to generate new transformant lines with all reporters at the same chromosomal location [21]. We refer to the line bearing a fusion of MSE3 and MSE2 without “spacer” as M32, with “spacer” as M3_2, a reverse-order fusion without “spacer” as M23, and reverse-order with “spacer” as M2_3 (Figure 1B and 1E). Site-specific transgenesis permits precise comparisons between multiple transgenic constructs by eliminating position effect (see Figure S1). We used previously published procedures to obtain quantitative gene expression data at nuclear resolution in space and 6.5 minute resolution in time [8], [16], [22], [23], and Figure 1C of this work. These data provide the relative expression levels of the reporter and eight TFs to 5–10% accuracy in each nucleus [6].

These data allowed us to make eight novel observations (Figure 1D). First, all four fusion enhancers do not drive the early broad expression seen in the native *eve* gene [6] and 1.7 kb proximal *eve* (1700) promoter [8], [24]. Second, overall expression levels of the four constructs decline after time class 6 (T6; 175.1 min AED). The next six features were seen in T6. In M32 the stripe 2 expression level increases by a factor of 3.5 compared with M3_2. In addition to the enhanced level of stripe 2 expression, the interstripe region between stripes 2 and 3 is derepressed in M32 compared with M3_2, causing a fusion of the two stripes. Peak stripe 7 expression is increased by a factor of two in M32 compared with M3_2. The positions of the peaks of stripes 2 and 7 are the same in M32 and M3_2. In contrast to stripes 2 and 7, there is a reduction by a factor of 0.7 in stripe 3 expression in M32 compared to M3_2. Finally, stripe 2 expression decreases by a factor of 0.2 and stripe 3 expression increases slightly in M23 compared with M2_3.

### A theoretical model of transcription

We employ a theoretical model that is intermediate between a content-based picture in which only the number of binding sites for each factor in an enhancer is significant [25], and, on the other hand, a grammar-based approach in which a precise arrangement of binding sites is required for regulatory function [26]. In our model, the physical arrangement of binding sites is quite important, but it is specified by rules that are sufficiently flexible to permit many solutions, reflecting the observed variability in binding site arrangement. We applied four design principles to formulate the model. First, we incorporated numerical implementations of a minimal set of regulatory mechanisms that are essential for the transcriptional control of the *eve* stripes 2, 3 and 7. Second, we designed the model in such a way that the mechanisms operate simultaneously. Third, the mechanisms are nonetheless separable, removable, and non-exclusive so that the relative contributions of each mechanism can be visualized as can the consequences of removing a specific mechanism *in silico*. Fourth, we performed a full statistical thermodynamics calculation to find the fractional occupancy of each binding site. Dynamic programming approaches are more computationally efficient but calculate summed fractional occupancies [14], [15]. Calculating with the the fractional occupancies of individual binding sites rather than their sum allows us to determine the contribution of each TF, binding site, and even nucleotide to gene expression.

**Figure 2:**
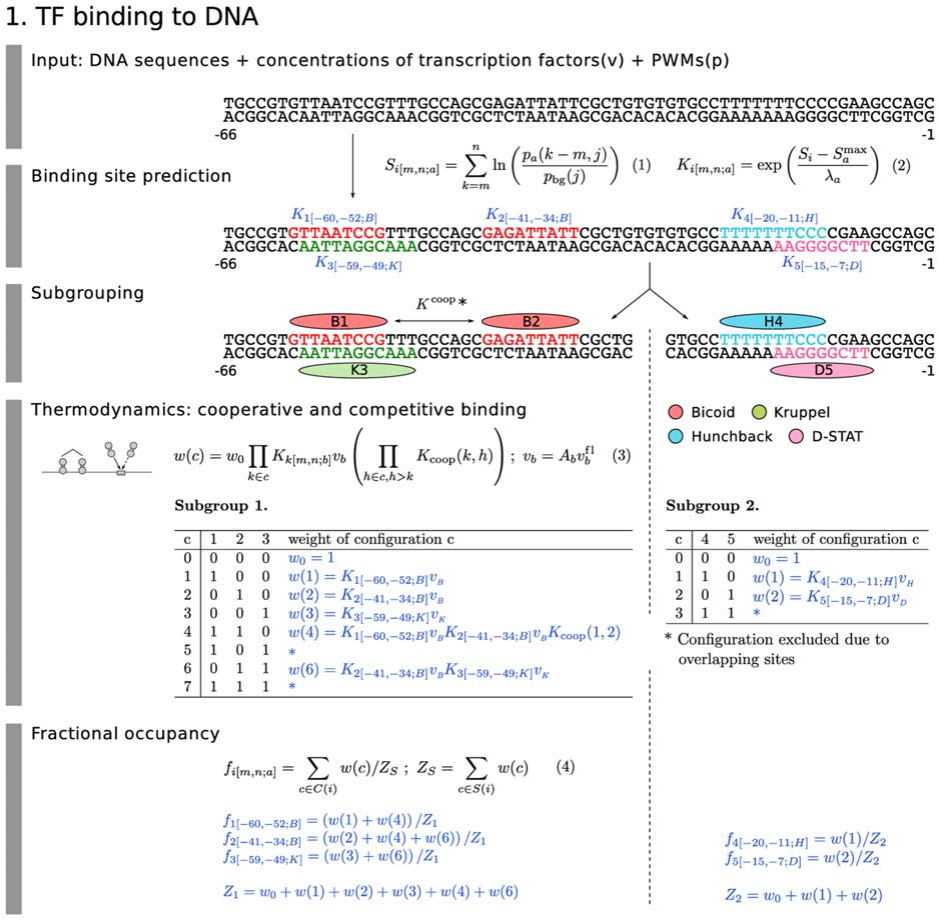
Model equations: TF binding to DNA. The model equations for binding site prediction (Equation 1 and 2), cooperative and competitive binding (Equation 3) and fractional occupancy calculation (Equation 4) are shown together in a flow diagram with cartoons of each mechanism on the left and an example application in blue with 5 TF binding sites. Subgrouping process partitioning the binding sites into independent binding groups allows faster computation without losing accuracy. In the example, we set the range of quenching to 20 bp.

The central players of transcriptional regulation are sequence-specific TFs that bind to DNA. The position of a TF binding site and its binding affinity are determined by a frequency matrix normalized to a position weight matrix (PWM; Figure 2, Equation 1). In this equation, 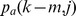 is the probability of finding base 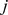 (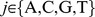) at the 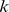
th position of a possible binding site for ligand 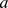 that extends from base 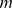 on the 5′ side to base 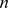 on the 3′ side, and 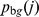 is the expected frequency of base 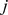 in *D. melanogaster*. When convolved with sequence, the score 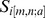 of the PWM on the sequence is proportional to the free energy of binding [27], and can be exponentiated to obtain the binding affinity 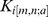 of ligand 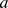 at site 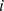. This is shown in Figure 2, Equation 2, where 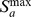 is the maximum possible score and 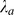 is the proportionality constant to free energy. We include a binding site in a calculation when its score is above a certain threshold. This threshold can be determined with different degrees of accuracy for each TF depending on the quality of the data used to construct its PWM (Materials and Methods).

In order to calculate the fractional occupancy 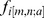 of TF 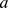 bound at a site 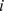 that extends between 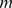 and 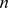 bp from the TSS, it is useful to first determine the effects of interacting configurations 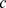 of TFs in terms of their weights 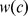 (Figure 2, Equation 3). These weights depend on TF concentrations 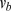, which in our dataset are in units of relative fluorescence 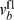 from confocal scans. To convert to true concentration units we multiply by a free parameter 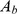 to obtain 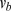. There are two types of interacting configurations. Some TF binding sites overlap or are closely placed. Overlapping sites lead to competitive binding by steric hindrance. We implement this phenomenon whenever sites overlap based on their physical size. We take a binding site to be at least 14 bp, the average size of a footprinted Bcd site. Footprinted data for Gt indicates a binding site size of 24 bp, a value used for this TF alone.

A second type of interaction has the opposite effect. Two adjacent sites may support cooperative binding, in which the free energy of binding of two simultaneously bound factors is greater than the sum of the free energies of them each binding separately [28], [19]. Transforming free energies to binding affinities, the nonadditive free energy term becomes a multiplicative factor 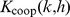, where 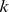 and 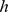 are two interacting binding sites (Figure 2, Equation 3). An important practical problem in the treatment of cooperative binding is the lack of experimental data concerning it for most TFs, particularly if heterologous cooperative binding involving two different proteins is allowed for. Considering all possible cooperative binding interactions would generate a combinatorial explosion of free parameters which are apt to give spurious results. In order to avoid this combinatorial explosion we implement cooperativity only when there is independent evidence for it, which is currently the case only for Bcd [30], [31]. Bcd cooperativity is also necessary to model the expression of the fusion constructs considered here. Without it, it was not possible to model the expression of M32 even in the presence of coactivation [32]. We model cooperative binding of Bcd by allowing the strongest Bcd binding site to interact cooperatively with the strongest remaining Bcd site within 60 bp (see Materials and Methods), and repeat these assignments with the remaining sites until all pairwise cooperative interactions are assigned.

With these mechanisms in hand, we use the concentration of TF 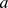 and other competing or cooperating TFs to calculate the fractional occupancy 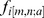 (Figure 2, Equation 4). We do this by summing the weights 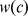 for all configurations 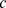 in which site 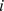 is occupied by 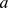. We then normalize against the sum 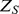 of all weights 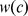 in group 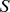, ensuring that for each site 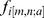 is between 0 and 1. As shown in the example associated with Equations 3 and 4 of Figure 2, each interacting group can be treated independently. We remark that the quantities 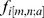 are fully deterministic intensive thermodynamic variables akin to concentrations. Although frequently derived from statistical mechanics [29] or even the Chemical Master Equation [33], they can also be derived from elementary considerations of equilibrium and stoichiometry [34]. Although 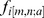 is frequently interpreted as the probability of finding ligand 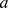 bound at site 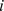, it is more accurate to view this quantity as the time averaged occupancy of site 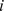 by 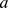. We thus assume that the binding states of the TFs that we explicitly consider equilibrate quickly compared to the time scale of changes in gene expression.

Once we have calculated 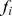, we calculate the effects of protein-protein interactions. A TF 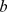 bound at site 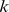 acting on a TF bound at site 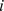 by mechanism 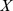 will be characterized by a parameter 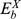 between 0 and 1 denoting the strength of 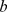
's action and a function 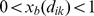 of the distance in bases between sites 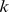 and 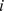 which controls the range at which the mechanism acts. The equations representing each mechanism are written such that they have the property that biological function can reside in multiple binding sites. We classify TFs as repressors or activators based on independent experiments. In what follows, 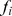 with no superscript denotes the physical fractional occupancy of site 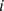. We write 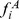 to denote the fractional occupancy of an activator and 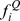 to denote the fractional occupancy of a quencher. We then allow for the possibility of “coactivation”, in which a repressor is transformed to an activator by the binding of a coactivator nearby. There is independent evidence that Bcd coactivates Hb in this manner [18], [35], as does Cad (see Materials and Methods).

**Figure 3:**
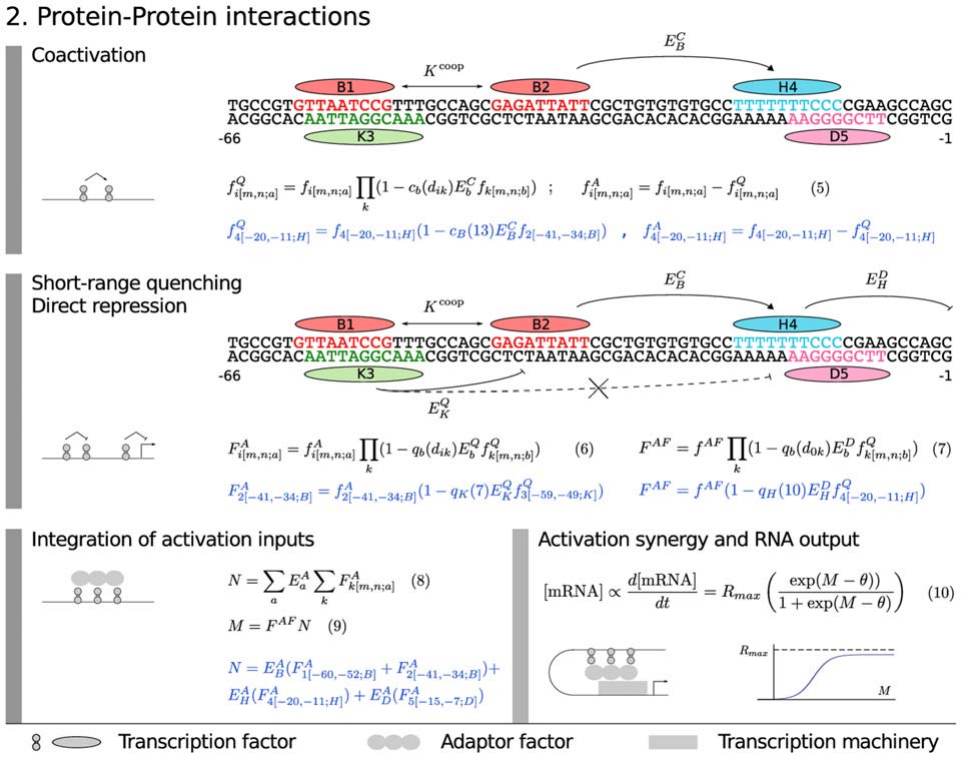
Model equations: protein–protein interactions. The model equations for coactivation (Equation 5), short-range quenching (Equation 6), direct repression (Equation 7), adaptor factor recruitment (Equation 8 and 9) and activation synergy (Equation 10) are shown together in a flow diagram with cartoons of each mechanism on the left and an example application in blue with 5 TF binding sites.

We represent coactivation as shown in Figure 3, Equation 5, where 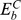 represents the coactivation efficiency of a coactivator 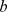 and the dependence of coactivation on distance is given by 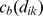. We constrain the activating and repressing activity of a coactivation target to sum to the physical fractional occupancy. The gap genes are short range repressors that act when bound within 150 bp of activators [36]–[38], a fact that we represent by convolving the fractional occupancies of all activators 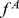 with those of quenchers 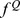 as shown in Figure 3, Equation 6 to obtain activator fractional occupancies 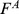 corrected for quenching, where 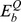 represents the repressive strength of TF 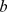 and the function 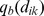 represents its range of action (Figure S2). When quenchers are bound within quenching range of the TSS they can prevent activators from acting at any range, a phenomenon described by Arnosti and coworkers as direct repression [36], [38]. Although longer range interactions of repressors with the TSS have been referred to as “direct repression” [39], [40], we limit ourselves to the short range interaction of Arnosti. This form of direct repression is represented in the model (Figure 3, Equation 7) in the same way as Equation 6 except that 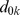 in this equation is the distance between the repressor binding site 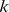 and TSS, and that the repressor does not act on 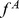 but on 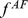. 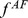 is associated with the transcription machinery that binds to the TSS, as we now describe.

With respect to activation, it is now clear that in metazoa activators do not directly contact the transcription machinery as they apparently do in yeast [41]. Instead, proteins that bind to TFs such as Mediator [42], [43] serve as a functional bridge between TFs and the basal machinery. These proteins are referred to as “adapter factors” (AFs) here following Guarente and Tjian [44]–[46]. Although AFs are sometimes referred to as “corepressors” or “coactivators”, we reserve that terminology in this work to TFs that bind DNA specifically. We view initiation of transcription as an enzymatic process catalyzed by AFs bound to TFs [4]. In the fly blastoderm, some AFs have been identified [43], [47], [48] and they are uniformly expressed from maternal mRNA, enabling us in this work to formulate AF action in a coarse-grained manner such that AFs are represented by a single composite chemical species whose fractional occupancy of binding to DNA bound TFs is given by 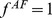 (Figure 3, Equation 7). Functionally active activators 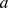 recruit the AFs with different recruiting strengths 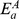 (Figure 3, Equation 8). Activators can act anywhere between the TSS and an insulator element, so here we do not need to consider 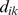, but simply sum the effects of the activators to obtain 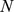, which is then corrected for the effects of direct repression to obtain 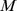 (Figure 3, Equations 7 and 9). The adapters then catalyze transcriptional initiation by decreasing an activation energy barrier 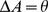 by an increment 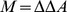. We describe the effect of lowering this activation energy by a diffusion limited Arrhenius rate law (Figure 3, Equation 10 and Materials and Methods). This rate law is exponential for a certain range of 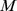, providing the capability to represent greater than multiplicative synergy between activators [49]. As the activation energy barrier falls to zero, the transcription rate 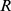 approaches 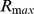 because diffusion of new polymerase molecules to the basal complex becomes rate limiting.

**Figure 4:**
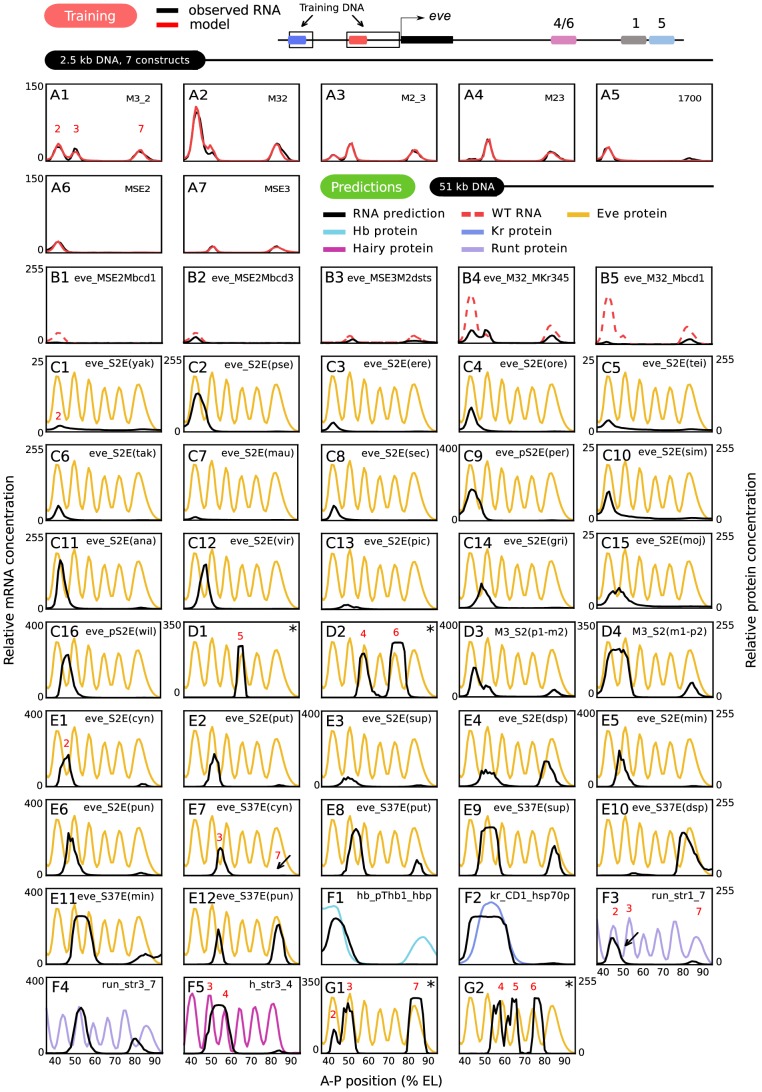
Training and predictions. (A) Training results for 7 constructs. RNA levels and model results are as shown in the key; the model result trace obscures the data in regions where both are superimposed. The regions of the *eve* locus used to generate the training data are indicated schematically. (B–G) Predictions of gene expression driven by DNA sequences that were not used for training. The sequences used are fully described in Table S4. Black lines are predicted RNA expression and colored lines are quantitative protein profiles of the corresponding endogenous loci. The scale of relative fluorescence levels for RNA is shown at the left of graphs, that for proteins on the right. All protein patterns are taken from the FlyEx database (http://urchin.spbcas.ru/flyex) [7]. An asterisk on a panel indicates the prediction was not made from model 6: D1-2 are from model 2, G1 is from model 7, and G2 is from model 1. See text, Figure S4 and Table S1 for details. (B) 5 mutant *eve* enhancers, described fully in the main text. (C) Stripe 2 enhancers from 16 different *Drosophila* species, with abbreviations and panel numbers (see Table S5 for full species name). The enhancer from *D. persimilis* (per), *D. grimshawi* (gri), *D. mojavensis* (moj) and *willistoni* (wil) was first identified in this study. (D) Other *D. melanogaster eve* enhancers. (D1) Stripe 5 enhancer. (D2) Stripe 4/6 enhancer. (D3) *pseudoobscura*- *melanogaster* stripe 2 chimera(p1-m2). (D4) *melanogaster*- *pseudoobscura* stripe 2 chimera(m1-p2). (E) Stripe 2 (S2E; E1–E6) and stripe 3/7 (S37E, E7–E12) enhancers from 6 Sepsid species, with abbreviations (see Table S5). (F) 5 non- *eve* enhancers from the *D. melanogaster* genes *hb* (F1), *Kr* (F2), *run* (F3-4), and *h* (F5). (G) Large 5′ (G1) and 3′ (G2) *eve* regulatory DNAs that contain multiple enhancers.

We fit the model described above to *lac*Z expression driven by the four fusion constructs shown in Figure 1 together with three additional fragments of the *eve* promoter, MSE2, MSE3, and 1700 during T6 (Figure 4A); fits were also performed to the four fusion constructs without the additional fragments (Figure S3). Inclusion of the three additional P-element constructs improved the predictive power of the model at the cost of one additional free position effect scaling parameter for each construct. Our TF dataset contains all of the factors essential for *eve* regulation in a region extending from the 1–2 *eve* interstripe to a position just posterior of stripe 7 (35% to 92% EL); additional TFs act on *eve* in the head and tail regions. These data constituted 406 independent observations of transcription rate corresponding to 58 combinations of nine TF concentrations acting on 7 constructs in each time class. The activators are Bcd, Cad, *Drosophila*-STAT (D-STAT), and Dichaete. The repressors are Kr, Kni, Gt, Tailless (Tll), and Hb. Of these, Hb was subject to coactivation by Bcd [20] or Cad, and hence it also functions as an activator. Independent experimental data (Text S1) allowed us to define binding thresholds for Hb and Bcd unambiguously, but in the case of other TFs these data implied a range of values for PWM thresholds and we allowed the threshold to be a free parameter within this range.

In addition to the 10 free parameters connected with position effect and PWM threshold, each TF 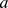 is associated with the parameter 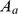 that scales the observed fluorescence units 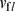 to absolute concentration units 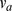 (Figure 2, Equation 3) as well as the parameter 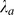 that scales the weight matrix score to units of free energy (Figure 2, Equation 2). Other parameters depend on the nature of the TF. Each activator is associated with an activation efficiency 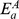, and each repressor to quenching and direct repression efficiencies 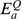 and 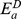 respectively. Thus each activator and repressor are associated with three and four parameters respectively. In addition, Bcd has a free parameter 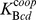. All elements of 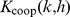 from Figure 2, Equation 3 are equal to 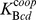 or unity. Both Bcd and Cad have free parameters 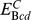 and 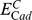 for the coactivation of Hb, and coactivated Hb has an activation efficiency 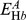. The activation energy barrier of transcription, 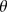, was also fitted (Figure 3, Equation 10). Finally, we fit the range of Cad and Bcd coactivation of Hb within a range set by independent experimental criteria (Figure S2 and Materials and Methods). Thus, 49 free parameters are fit to 406 observations.

### Model training and validation

Multiple fits to the training data resulted in a group of models driving essentially identical expression patterns (Figure S4) and having similar but not identical parameter values (Table S1). The models resulting from the fitting procedure agree with experimental data within the limits of experimental accuracy with two very small exceptions (Figure 4A and Figure S4). First, the peak of stripe 3 in M32 is one nucleus anterior with twice the expression level in the model compared to data (Figure 4A2). Second, stripe 7 expression in the 1700 construct is almost absent in the model (Figure 4A5). It is an important validation of our approach that we can numerically represent the effects of these enhancer fusions at this stringent level of precision.

An even more stringent test is to examine the predictive power of the model on DNA sequences not used for training. We tested the predictive power of the model on 6 classes of regulatory DNA that are thought to be largely regulated by the same maternal and gap genes used in the training set. These are 1) 5 mutant *eve* enhancers; 2) Stripe 2 enhancers from 16 Drosophilid species; 3) The *melanogaster eve* stripe 4/6 and stripe 5 enhancers and two *melanogaster-pseudoobscura eve* chimeric stripe 2 enhancers; 4) 12 enhancers from six Sepsid species; 5) Fifteen enhancers from four gap genes and two primary pair-rule genes other than *eve*; and 6) Large upstream and downstream *eve* regulatory DNAs that contain multiple enhancers. Each DNA sequence tested contained one or more enhancers and basal promoter sequence. If the basal promoter sequence for an enhancer construct was not known, *eve* basal promoter sequence was used. Except as noted, all predictions shown in Figure 4 were made from model 6 (Figure S4 and Table S1) with no alterations of any parameter except the sequence itself. If a prediction from a parameter set other than model 6 is shown in Figure 4, the corresponding prediction from model 6 is shown in Figure S5. Altogether we tested 54 sequences amounting to 62 kb of DNA, and obtained good predictions for 44 sequences driven by 51 kb of DNA, as we now describe.

The classic literature describing the 5′ regulatory region of the *eve* locus contains numerous studies of the effects of very small site-directed mutations affecting only 2 to 6 bases. Our ability to predict the effects of such mutations is of interest not only for checking the validity of the model, but also has implications for the interpretation of single base pair polymorphisms (SNPs) and small indels. Here we consider a 3 base pair change in the bcd-1 site (Mbcd-1) in the context of both MSE2 and M32, a 5 base pair change in the bcd-3 site (Mbcd-3) [24], a two base pair change in each of two D-STAT sites (M2dsts) [50], and changes of 5, 3, and 6 base pairs respectively in the Kr-3, Kr-4, and Kr-5 sites (MKr345) [20]. The model correctly predicts that Mbcd-1 causes a larger diminution of expression than Mbcd-3 (Figure 4B1-2 of this work; cf. Figure 6D and F in [24]). The model's prediction of greatly diminished expression in M2dsts is qualitatively correct, but experiment indicates a complete abolition of expression (Figure 4B3 of this work; cf. Figure 8D in [50]). The prediction of reduced but equivalent expression of stripes 2,3, and 7 while 2 and 3 remain fused when MKr345 is placed in M32 is completely correct (Figure 4B4), and we correctly predict the restoration of stripe 2 expression in the presence of a non-functional bcd-1 site when Mbcd-1 is placed in M32 (compare Figure 4B1 and 4B5), but the model predicts that stripe 3 is absent when in fact it is reduced (Figure 4A and 4C for MKr345 and Mbcd1 respectively in [20]).

We confronted the model with DNA sequence from the stripe 2 enhancers of 16 *Drosophila* species other than *melanogaster* (Figure 4C1-16), four of which were first identified in this study (Figure 4C9, 4C14-16). In ten cases, stripe 2 expression was coextensive with the *melanogaster* stripe pattern (Figure 4C1-10). There is experimental evidence that *D. yakuba, D. pseudoobscura, and D. erecta* stripe 2 enhancers express coextensively with the *melanogaster* stripe 2 (Figure 4C1-3 of this work; cf. Figure 6 in [51]). Our results are in substantial agreement with these findings, up to a posterior shift of about one nucleus in *pseudoobscura* and *erecta* (Figure 4C2-3). To our knowledge, no experimental observations have yet been made of the positions of stripe 2 driven by the remaining 13 *Drosophila* stripe 2 enhancers in *D. melanogaster*.

As an initial test of the model's predictive power on sequences with no homology to those used in training, we found that we can correctly predict expression of *eve* stripe 5 and stripes 4 and 6 from their respective enhancers (Figure 4D1-2 from model 2; see Figure S5A–S5B for model 6 results; cf. Figure 2B and 2D in [52]). We then extended this test to interspecific chimeras. Altered expression patterns driven by chimeric constructs with half of the stripe 2 enhancer from *pseudoobscura* and and half from *melanogaster* have been observed by enzymatic assays (Figure 1i and 1l in [53]). With the *melanogaster* sequences on the 3′ end, a posterior expansion of stripe 2 was described, which appears to extend to a variable fusion of the two stripes and a reduction in stripe 3 amplitude; our model predicts a partial fusion and a reduction in the amplitude of stripe 3 (Figure 4D3). The complementary chimeric enhancer drives a fusion of stripes 2 and 3 which is also predicted by the model (Figure 4D4) [53].

We also made predictions of expression patterns driven by regulatory sequences from the *eve* locus of six species of Sepsid flies. These species are about twice as evolutionarily distant from *D. melanogaster* as *D. melanogaster* is from the most distantly related *Drosophila* [54]. Our model, when challenged by Sepsidae DNA, predicts stripe 2, 3 and 7 expression driven by the corresponding Sepsid enhancers (Figure 4E1-12). Some of these predictions are confirmed (Figure 4E1-3 and 4E7-9). Stripe 2 and 3/7 enhancers from *T. cynipsea, T. putris* and *S. superba* have been tested for expression in *D. melanogaster* and shown to express *eve* stripes 2, 3, and 7 (Figure 5B, C, E and F in [54]); these are correctly predicted with the single exception of a failure to correctly predict observed stripe 7 expression driven by the *cynipsea* 3/7 enhancer (Figure 4E7, arrow). The model predicts that the Sepsid stripe 2 enhancers drive stripe 7 expression at levels which vary from species to species (Figure 4E1-6). It is confirmed experimentally that 78% of embryos containing the *S.cynipsea* enhancer and 55% of embryos containing the *T. putris* enhancer appear to have stripe 7 expression [54]. The model also predicts that stripe 2 expression from *S.cynipsea* and *T. putris* is shifted to the posterior (Figure 4E1-2) and that the shift is larger in *T. putris*, a point supported by published observations (Table 2 in [54]). These observations, based on visual observations of enzymatically stained embryos, indicate that the posterior borders of gene expression driven by *S. cyn* and *T. put* S2Es are shifted 2% and 3% EL posterior respectively, with a reported uncertainty of about 1% EL. Our model predicts shifts of 4% and 9% EL if the posterior border is taken to be the position of half maximum expression. It is notable that our model predicts stripe 3 and 7 activity from the putative stripe 2 enhancer of *Dicranosepsis sp.* (Figure 4E4), and further predicts that in a *D. melanogaster* context this species' putative 3/7 enhancer drives stripe 7 expression at levels an order of magnitude greater than the maximum level of stripe 3 expression (Figure 4E10).

A more stringent test of the model is to predict the expression driven by the enhancers of *D. melanogaster* genes other than *eve*. Not all such reported enhancers can be tested, as some require TFs (such as pair-rule gene products) not considered in this study. We tested 15 enhancers of gap and pair-rule genes using the same TFs as were employed for the training set. Among the gap genes, we obtained correct predictions for expression driven by the pThb enhancer of *hb* (Figure 4F1 of this work; cf. Figure 1 in [55]) and the CD1 enhancer of *Kr* (Figure 4F2 of this work; cf. Figure 5a in [56]). With respect to the Runt 1_7 and 3_7 enhancers (Figure 4F3-4 of this work; cf. Figure 3K and 3D in [57]), we correctly predict the expression of *run* stripe 3 and reduced expression of *run* stripe 7 compared to stripe 3, although in Runt 1_7 the predicted stripe 1 is coextensive with stripe 2 of the *run* protein pattern. The predicted pattern of *run* stripe 7 is shifted about 2 and 7 nuclei to the anterior of the native *run* stripe in Runt 1_7 and Runt 3_7 respectively. The predicted pattern of the h_str3_4 enhancer (Figure 4F5) is correct, as this enhancer drives an expression domain that does not contain the *h* 3–4 interstripe (Figure 4C in [58]). Ten additional enhancers from the genes *hb*, *kni*, *gt*, *run*, and *h* gave incorrect predictions (Figure S5C–S5L). In each case, expression in the correct domain was absent although in some instances small amounts of ectopic expression remained.

Our model is not limited to experimentally isolated enhancers, and so we attempted to predict expression driven by the approximately 4 kb of 5′ and 3′ noncoding DNA which respectively control stripes 2, 3, and 7 (Figure 4G1, parameters from model 7; see Figure S5M for model 6 prediction; cf. Figure 1G in [59]) and stripes 4, 5, and 6 (Figure 4G2, parameters from model 1; see Figure S5N for model 6 prediction; cf. Figure 4I in [60]). Our initial prediction was completely incorrect, showing saturated blocks of expression without interstripes. When the threshold 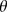 was increased by hand, we obtained the qualitatively correct predictions shown in Figure 4G1-2. Although requiring the hand tuning of a single parameter, we consider it highly significant that the predictive power of the model extends beyond single enhancers discovered by *in vivo* assays.

### Functional analysis of the fusion gene expression

The accurate modeling of expression from fusion constructs together with correct predictions of expression patterns not used in training provide evidence that the model captures the underlying rules governing *eve* transcription. Given this level of credibility, it is also possible to use the model to understand how the interplay of multiple transcriptional mechanisms give rise to the very complex expression changes induced by removing the “spacer” DNA.

**Figure 5:**
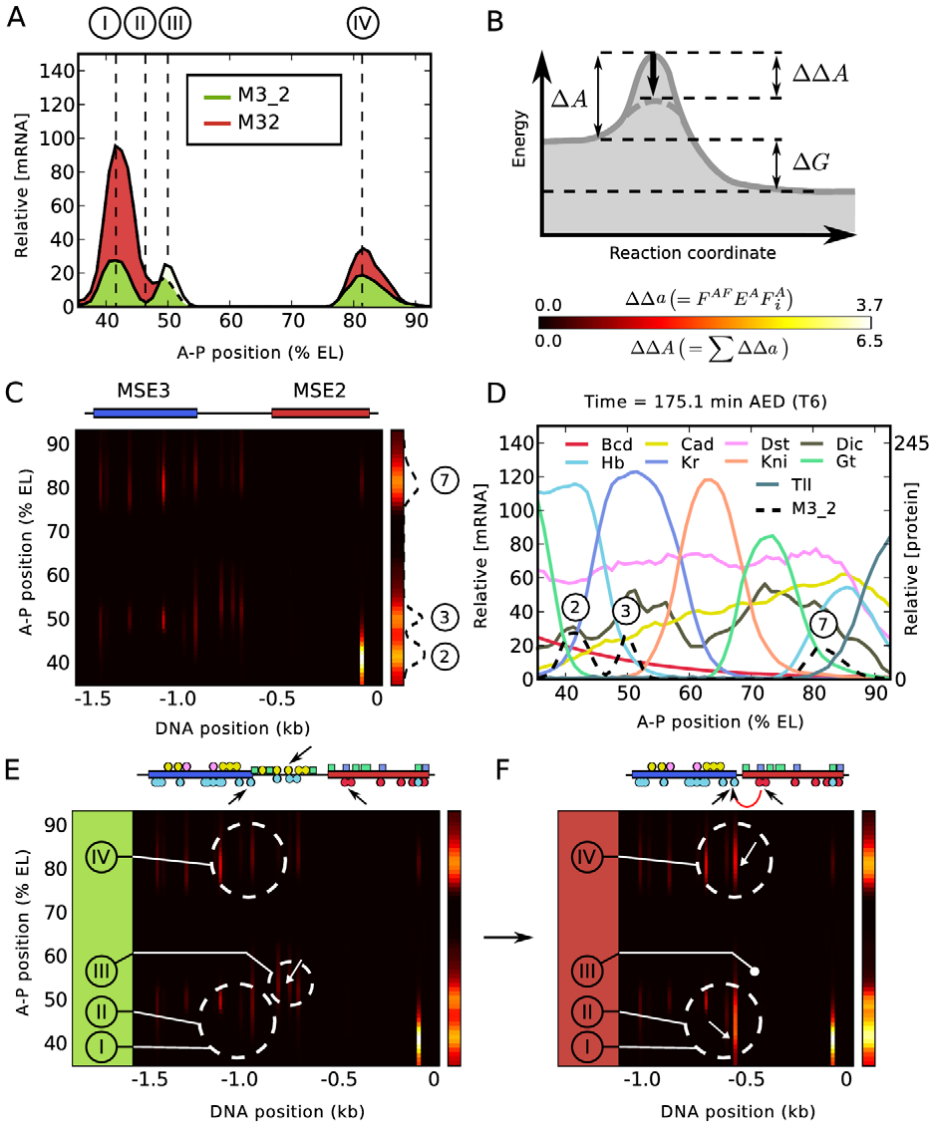
Regulatory analysis of M3_2 and M32. (A) The expression profiles driven by M3_2 and M32 are subdivided into four distinct zones I to IV for analysis as shown. Two additional zones V and VI involving expression changes between M2_3 and M23 are shown in Figure S7. (B) Illustration of a catalyzed reaction with free energy change 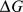 and activation energy barrier 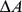. Catalysis by activators reduces the barrier by 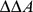. A scale bar of two heatmaps used in (C), (E), and (F) is shown. The 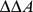 heatmap applies to the vertical bars on the right hand side of these panels and the 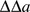 heatmap applies to the square panels in (C), (E), and (F). 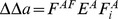; compare with Equations 8 and 9 in Figure 3. (C) Distribution of activation energy barrier changes at single binding site resolution for M3_2 as a function of A-P position on the embryo and number of basepairs 5′ to the M3_2 TSS. The positions of MSE2 and MSE3 are schematically shown at the top. 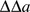 for each activator binding site is shown in the central panel according to the key in (B) and the summed activation 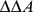 in the right hand bar. Peaks of activation corresponding to stripes 2, 3, and 7 are indicated. (D) Expression levels of RNA expression driven by M3_2 together with regulating TFs at cellular resolution, as shown in the key. In the key, standard abbreviations are used except that Dst indicates D-STAT and Dic indicates Dichaete. (E) and (F) show a regulatory dissection of expression changes induced by removal of the “spacer” with activation represented as in (C). Selected binding sites for M3_2 and M32 are shown at the top of (E) and (F) respectively, with TF specificity indicated by color as shown in the key for (D). The full set of binding sites is shown in Figure S6. The black arrows show binding sites involved in coactivation; the red arrow in (F) indicates the major coactivation interaction in M32. Circled areas indicate groups of binding sites critical for expression changes in different zones as described in the text.

The fusions introduce six types of quantitative alterations in expression, each of which occurs in a small spatial region containing 2–3 nuclei, which we call a “zone” (Figure 5A). With respect to the M32 fusion compared to M3_2, in zone I stripe 2 expression is increased by a factor of almost four; in zone II the 2–3 interstripe is derepressed; in zone III stripe 3 expression is reduced; and in zone IV stripe 7 expression is increased. With respect to the M23 fusion compared to M2_3, in zone V stripe 2 expression is reduced and in zone VI stripe 3 expression is slightly increased (Figure S7A). We analyzed the causes of these effects by plotting the contributions to the activation 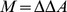 (Figure 5B) as a function of position on the A-P axis and the regulatory sequence (Figure 5C), where each position on the A-P axis defines a unique set of TF concentrations as shown in Figure 5D. Annotating these diagrams with the identity of key binding sites and comparing activation in M32 and M3_2 indicates which TFs and binding sites lead to the effects observed Figure 5E–5F). These diagrams show that the major source of activation is from coactivated Hb bound at the hb-3 site by Bcd bound at the bcd-1,bcd-* and bcd-2 sites (Figure 5E–5F and ). With respect to zone I, we found that the increase of gene expression is almost entirely the result of coactivation of two sites of bound Hb by Bcd. It occurs because of the deletion of the “spacer” DNA between MSE3 and MSE2, which reduces the distance between the two Bcd sites in MSE2 and the two Hb sites in MSE3 from more than 400 bp to about 150 bp, permitting coactivation (Figure 5E–5F, lower black arrows; Figure 5F, white arrow).

These two Hb sites extend about 60 bp into MSE3, about 15% of its total length. These Hb sites are subject to repression by quenchers bound within 150 bp on their 5′ side, including one site for Gt (Figure S6). Thus, the same functional interactions characteristic of MSE2 now extend 200 bp into MSE3, about 40% of its length. These points indicate that in M32, 40% of MSE3 has been recruited to be a functional part of MSE2. This functional recruitment includes the setting of the anterior border of stripe 2 by repression. The location of the anterior border of stripe 2 is unchanged in M32 compared to M3_2 based on the location of half maximum expression, despite the synergistic activation by Bcd and Hb, whose concentrations are essentially equivalent at the peak of the augmented stripe 2 and at its anterior border. A single Gt binding site in MSE3 together with a single site in the stripe 2 enhancer are sufficient to repress anterior expression driven by the recruited portion of MSE3. Such robustness in border control would be impossible if repression were to occur only by steric competition. These results also demonstrate that the borders of enhancers are not intrinsic, but instead are determined by genomic context. In zone II, the derepression of the interstripe is a consequence of the fact that Kr binding sites are predominantly distributed on the 3′ end of MSE2, close to the hb-3 site (Figure S6A). There is a single Kr binding site (Kr-5) within range of the coactivated Hb bound to MSE3, and it is insufficient to provide complete repression in zone II.

The expression changes that occur in zones III and IV are connected with the fact the “spacer” in M3_2 is in fact a functional component of the 3/7 enhancer. The reduction of stripe 3 expression levels in zone III is not recapitulated by fitting the model to the full set of seven constructs (Figure 5A), but is found in fits made only to the four fusion constructs (Figure S3A). The cause of the change in expression in zone III is in either case evident from inspection of Figure 5E (downward pointing arrow and white arrow), which show that the “spacer” contains Hb binding sites which are coactivated by Cad, the removal of which decreases expression. There are, in addition, repressor sites in the “spacer” (Figure S6A). In zone IV, the model consistently gives a correct representation of the increase in stripe 7 expression in M32 compared to M3_2, and this is a consequence of the removal of repressor sites located in the “spacer”. The effects seen in zones III and IV are critically dependent on the precise balance between activation, coactivation, and repression. This leads to residual ambiguity in how models with differing training data and parameter sets account for expression changes in these zones, but in all analyses the “spacer” plays a major functional role and is not an inert segment of DNA.

The “spacer” DNA in M2_3 is a component of the full stripe 2 enhancer S2E [61], [62], and its removal causes a severe diminution of stripe 2 expression in zone V and a much smaller increase of stripe 3 expression in zone VI, with stripe 7 unaffected (Figure S7A). These effects occur because the M2_3 “spacer” DNA contains two Bcd and two Hb binding sites (Figure S6D and S7B). The strongest Bcd site in MSE2 is bcd-1, and in M2_3 it preferentially establishes pairwise cooperativity [31] with the next strongest site (bcd-(−1), Figure S6D), which is the most 5′ of the two sites on the “spacer”. In addition, a cooperative interaction exists between Bcd bound at the bcd-* (unnamed footprint site; see Figure S6D and Figure 4 in [18]) and bcd-2 sites. The net result is that in M2_3 these two pairs of cooperatively bound Bcd provide strong coactivation to two Hb sites, one of which is in the “spacer” (Figure S7B, zone V region and downward pointing white arrow). In M23, the absence of the “spacer” causes major rearrangements of pairwise cooperative interactions among bound Bcd molecules in MSE2 because bcd-(−1) is lost. Without the “spacer”, Bcd bound at bcd-1 cooperates with Bcd bound at bcd-*, while Bcd bound at bcd-2 cooperates with Bcd bound at bcd-3 (compare Figure S6E and S6F). This configuration of cooperative interactions results in a lower fractional occupancy of Bcd compared to that seen in M2_3. Although Hb sites at the 5′ end of MSE3 are recruited as a part of the stripe 2 enhancer by cooperatively bound molecules of Bcd in M23 (Figure S7C, white arrow), the net reduction in bound Bcd without the “spacer” causes a reduction of activation in zone V. The contrasting small increase in expression in zone VI happens because the “spacer” also contains Kr sites (Figure S6D) which are heavily bound in the *Kr* expression domain which contains *eve* stripe 3 (Figure 5C). It is this difference in Kr levels which causes the opposite effect in zone VI compared to zone V.

## Discussion

In the work described here we have gone beyond modeling only individual experimentally identified enhancers, and have done so at a level of resolution comparable to that required for organismal survival. Although our previous work with a version of this model not incorporating cooperativity or coactivation was comparably accurate and capable of representing stripe 7 expression driven by sequences outside of the 3/7 enhancer, the modeled DNA contained only one classical enhancer, S2E [4], [8]. In contrast, the expression data used in the present study not only involved two enhancers, but more importantly dealt with a situation in which the function of these enhancers was critically altered by juxtaposing them and thus altering their function. These rearrangements provided a powerful constraint on the possible rules of transcriptional control, as demonstrated by the prediction of expression patterns seen here. Finally, the model can be used as an analytic tool with which to understand how multiple transcriptional mechanisms operate simultaneously to produce observed patterns of expression.

Highly precise experimental data made this study possible, and their importance cannot be overemphasized. The inherent transcriptional machinery is exquisitely precise, and fundamental understanding of its functioning requires data at a cellular level of precision. Our dataset has that level of precision because we performed simultaneous staining of reporter-driven *lac*Z expression and native Eve protein, allowing us to register the reporter data with our full TF dataset [8]. The intrinsic variability of gene expression prevents such registration by measurements of the position of reporter expression alone. This point illuminates a problem regarding the current unbalanced state of technology in genomics. Sequence can be obtained readily and cheaply. Yet, the inability to monitor gene expression at cellular resolution in a high throughput manner together with a lack of understanding of the code for regulatory logic has in general limited genomic level investigations of regulatory DNA to statistical association studies. The work reported here was made possible by a high resolution dataset created over many years. Although the data was quantitated using high throughput methods, staining and microscopy were carried out manually.

The quality of fit to the training data indicates that the model is reasonably complete for the stripe 2 and 3 *eve* enhancers at the developmental time assayed. Previous attempts to model both stripes simultaneously failed, most probably because of a failure to incorporate coactivation of Hb by Bcd and Cad [14], [15]. Further support for the current model is afforded by its predictive capability. In *melanogaster*, we obtained accurate predictions for expression driven by the stripe 5 and 4_6 enhancers. We were also able to correctly predict the effects of site-directed mutations affecting only 2–6 base pairs. This result indicates that the model might ultimately have utility in predicting the effects of SNPs, a point with implications for both medicine and evolutionary biology.

With respect to stripes 2, 3, and 7 in non- *melanogaster* species there are no contradictions to available experimental results. This is a strong indication that we have captured major elements of the fundamental rules of transcription, as these diverged enhancers have considerable turnover in binding site composition [51], [53] among the Drosophilids and no homology except for short sequences involving overlapping binding sites in Sepsids [54]. In fact, enhancers from only 4 Drosophilid and 3 Sepsid species have been qualitatively assayed by transformation into *melanogaster*, so that we have furnished a rich set of quantitative predictions that can be examined in future experiments.

With respect to predictions of the expression of other *Drosophila* genes, we obtained good results for the *h* 3_4 and *run* 3_7 enhancers. The predicted *run* 1_7 enhancer pattern had better registration of stripe 7 with protein pattern than predicted for 3_7, with the strange result that the predicted pattern is in perfect alignment with *run* stripe 2 rather than stripe 1. This last prediction may be erroneous. Although we are aware of no published co-staining data of the *run* 1_7 enhancer with native *run* protein or RNA, such data exists for a larger segment of DNA which drives *run* stripes 1, 3, and 5 and contains *run* 3_7 [57]. With respect to gap genes, we have good agreement of predicted patterns for the *hb* pThb1 and *Kr* CD1 enhancers, but the agreement is poorer for other *Kr* and *hb* enhancers, *kni*, and *gt*. In the case of *gt*, the lack of expression in the native domain is a consequence of the presence of numerous Gt binding sites. There are indications that Gt has autoactivation activity [63]. It is possible that Gt has a coactivator on its own promoter that was not included in this study.

Although enhancers are frequently referred to as *cis*-regulatory “elements”, they are not elementary or fundamental objects. They are not elementary because they do not have well-defined boundaries. We demonstrated the context-dependent border of MSE2 in this study by showing that the increased level of stripe 2 expression in M32 was a consequence of the recruitment of 40% of MSE3 to become a functional component of MSE2. Moreover, MSE2 and S2E both drive stripe 2 and can rescue lethality [62], and MSE2 is not completely minimal in the sense that smaller regions of DNA within it can drive weak and variable stripe 2 expression [24], [61]. Enhancers are not functionally fundamental objects because most enhancers drive expression domains which are similar to but not identical with those driven by the intact locus. Complete fidelity requires additional sequences. With respect to *eve* stripe 3, this point has been evident for some time in mutant genotypes, although the additional sequences required are as yet unidentified (compare Figure 4B in [19] with Figure 5A in [64] and Figure 5B in [19]). In the case of *hb*, the lack of fidelity is evident in wild type and complete fidelity is restored by a shadow enhancer [3]. The real challenge in regulatory genomics is the prediction of expression from an entire locus.

Our ability to model expression of the fusion constructs and to predict expression of stripes 2, 3, and 7 driven by 5′ noncoding sequence and stripes 4, 5, and 6 by *eve* 3′ noncoding sequence demonstrates that the applicability of the model is not limited to previously identified enhancers. These results support an idea advanced by Gray, Levine, and coworkers that short range repression is required for the independent action of multiple enhancers [39]. Indeed, lines of evidence from both experiment [19], [24], [52], [61] and theory [8], [65] indicate that *eve* stripes are generated by repression from gap genes. Because gap gene expression domains are wider than *eve* stripes, silencing from these genes would result in a repressed region comparable in size to that of a gap domain and could not produce the observed stripes.

Our predictions of expression driven by large DNA segments are less clean than those of single enhancers in the sense that they required hand tuning of the threshold 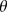 to prevent completely saturated expression domains comprising stripes 2–3 and 4–6 respectively. This saturation appears to involve a lack of balance between activators and repressors as the length of modeled DNA increases, but it is not possible at this time to distinguish between problems with the model and the training data. With respect to the model, this lack of balance may stem from the unlimited range of activators and the limited range of quenchers. In order to know whether this model property is biologically correct or incorrect, it is necessary to quantitatively determine how the amplitude of a given stripe changes as it is driven by larger DNA fragments. This point is not captured in our training data because only the four fusion constructs, all of similar total length, were transformed to a targeted site. Shorter and longer DNA fragments were not targeted transformants and hence required a free parameter scaling the amplitude to account for position effect. The quantitative characterization of expression driven by fragments of varying size transformed to a common chromosomal site is an important experimental task for future work. It will also be important to generate rescue constructs containing both native and *lac*Z message in order to standardize between observed levels of native and reporter transcripts. We believe that the results in this paper, while incomplete, demonstrate the feasibility of constructing a precise, quantitative, and predictive model of an entire locus that would also account for its enhancer structure.

We obtained multiple sets of parameters that fit the data well (Table S1 and Figure S4), indicating that the training data constrain but do not completely determine the parameters. The parameter sets in Table S1 give much more divergent behavior with respect to predictions than they do with the training data. We cannot eliminate the possibility that that full set of DNA sequences considered in Figure 4 cannot be described by the same set of parameters, indicating an underlying defect in the model. We believe that it is more likely that residual indeterminacy in the parameter set determined by the training data is the cause of divergent predictions. Just as an experimentalist devises a decisive experiment by careful arrangement of conditions, we think it likely that not all experimental data is equally suitable for training. For example, the model used here if trained on stripe 2 data only will not show coactivating activity for Bcd or Cad (data not shown). An important question for future work will be to apply ideas from statistics and machine learning theory to understand what constructs should be used so as constrain the model parameters as tightly as possible and/or decide whether the model is missing a particular regulatory mechanism.

A useful model not only has predictive power, but also explanatory power, a point illustrated by our analysis of the expression changes seen in zones I through VI. This power stems from the fact that we keep track of the fractional occupancy of each individual binding site. This level of resolution combined with the capability of removing a specific mechanism *in silico* allows us to assay the relative contributions of the multiple mechanisms of transcriptional control that operate simultaneously. Moreover, fractional occupancy in turn depends on affinity and hence DNA sequence, affording us a way to precisely characterize regulatory changes introduced at the level of individual base pairs. This analytic power, together with the importance of quantitative data, is well illustrated by considering questions raised in the classic study which first considered the fusions analyzed here [20]. In this work, which was instrumental in establishing the importance of spacing for correct enhancer function, the authors proposed that the diminution of expression in zone III was a consequence of *Kr* sites in MSE2 coming into repressive range of activator sites on MSE3. Small and coauthors supported their hypothesis by mutating the three footprint Kr sites on MSE2 and noting that these mutations resulted in an expression pattern in which stripes 2 and 3 were partially fused and of equal amplitudes, which were greater than that of stripe 7 (Figure 4A in [20]).

We found that the reduction of stripe 3 expression in zone III was a consequence of the removal of activator sites in the “spacer”. Furthermore, the model predicts that the equal amplitudes of stripes 2 and 3 in the mutations of the three footprint Kr sites are because of the fact that the increased stripe 2 expression levels driven by M32 were reduced by these mutations (Figure 4B4). This reduction in stripe 2 expression is a consequence of a reduction in the affinity of the bcd-5 site, which overlaps with the kr-5 site, by a factor of 5. This reduction in affinity was not predictable in the early 1990s when high quality PWMs for Bcd were unavailable.

Although an improvement on previous efforts, the work presented here does not constitute a complete solution to the problem of understanding *cis*-regulatory logic. In considering what may be required for further progress in understanding *cis*-regulatory logic, it is necessary to distinguish between limitations on available data and limitations of the model itself. It is significant that we were able to predict the expression of highly rearranged Sepsid enhancers up to the resolution of available data, while our results for gap and pair-rule enhancers other than *eve* in *melanogaster* were mixed. We believe that this is a consequence of the fact that some of these enhancers utilize TFs and perhaps interactions among the TFs that are not important for driving *eve* stripes 2, 3, and 7. One example is Dichaete, which was not considered in our initial efforts to model the fusions (data not shown), but was included in the training set reported here because it has been reported to be an activator of *eve* stripes 4,5 and 6 [66]. A possible example of a missing interaction is the spurious auto-repression of *gt* in its own expression domain (Figure S5F). Given that the expression training set used in this study was driven by only 2.5 kb of DNA from a single locus, it is likely that the use of a more diverse training set would result in improved predictions.

As regards the model, it is clearly incomplete in the sense that it does not contain a full set of regulatory mechanisms. As a basic point of model design, we incorporated a representation of a regulatory mechanism into the model only when there is specific evidence that it acts in the experimental system under consideration. This means that some mechanisms that are known to occur and are easy to represent mathematically, such as corepression [67], [68] and cooperative binding by heterologous pairs of proteins [69], were not incorporated in this study because there is no evidence that they occur in that portion of the *eve* control region used for the training set. With respect to cooperative binding to DNA, there is a pressing need for high-throughput quantitative data. Microfluidic methods provide a feasible way to address this problem [70].

A more fundamental issue concerns the role of chromatin structure, an area where new theoretical ideas are required. Silencing is thought to involve changes in chromatin structure. This phenomenon cannot be modeled simply by modifying the distance function 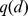 for short range repression because such a modification cannot account for radical changes in the range of silencing observed when the number of silencer binding sites is altered [71]. It is possible that the way forward involves spreading inactivation models of the type proposed by Sengupta [72]. A critical unsolved problem is the incorporation of regulators into such models, and the study of so-called chromatin marks may be useful in this regard.

The *eve* locus itself may prove a useful system in which to pursue such studies. The proximal 1.7 kb of 5′ noncoding DNA from the *eve* gene drives a pattern of expression in cleavage cycle 13 and the first 6 minutes of cleavage cycle 14A that closely resembles that of the entire locus [6], [8]. In contrast, the fusion constructs considered here do not express at these early stages (Figure 1D), nor does MSE2 (data not shown). Moreover, changes of expression occur after T6 that suggest early signs of the midblastula transition. These changes take the form of decreases of expression in stripes 3 and 7 by T8, together with a loss of registration with the native *eve* pattern caused by the fact that reporter expression does not follow the anterior shifts observed in expression driven by the native locus [6]. It is possible that these changes of chromatin state can be probed in a manner that will suggest new theoretical ideas by conducting ChIP-seq or hypersensitivity studies on embryos prepared with extremely high temporal resolution.

In conclusion, our model demonstrated that short-range quenching and coactivation are essential mechanisms conferring independent action of enhancers in the large *even-skipped* regulatory DNA. We found no decisive evidence that the length scales over which these interactions occur are fundamentally different. Short range quenching had a length scale of 150 bp, set from published experiments. The length scale of coactivation of Hb by Bcd was almost exactly the same (Table S1), despite it being allowed to vary in the fitting procedure. These mechanisms are clearly necessary for understanding the regulation of the entire *eve* locus, and establishing their sufficiency will be the subject of future work. In the case of both mechanisms we expect that better knowledge of phenomenology would lead to superior understanding. For example, Arnosti's group has produced greatly improved data on short range repression that suggests periodic behavior in limitations exist not only for the data but also for the model [13]. Alternatively, it might be more useful to reduce the number of parameters by constraining the range and functional form of all short range interactions to be identical. Such a choice would reflect a picture in which the scale of all short range interactions are set by the length of DNA associated with a single nucleosome (160–240 bp) [73]. Fixing this length scale based on structural considerations would connect our model with an important body of data.

Our predictions of expression patterns from many Drosophilidae and Sepsidae strongly suggest that the fundamental rules of metazoan transcription are well conserved over the course of evolution. As a syncytium, the *Drosophila* blastoderm is very specialized as a developmental system but there is no reason to think that transcription in this system operates differently than in the rest of the metazoa. As yet there are two barriers that must be crossed to establish a general theory of eukaryotic transcriptional control. One is experimental—training data require not only expression levels and regulatory sequence, but also the concentrations of TFs. Another is theoretical—a framework is needed to understand long range interactions in the chromatin.

## Materials and Methods

### Construction of fusion reporters

The M32, M3_2, M23, and M2_3 transformant lines were generated by excising the *Eco*RI- *Xba*I fragments from four *eve*- *lac*Z pCaSpeR plasmids [20] and ligating them into the RMCE (Recombinase Mediated Cassette Exchange) vector pBS(KS+)- *lox*- *white*- *lox2272* [21] cut with *Eco*RI and *Spe*I. Each *Eco*RI- *Xba*I fragment contained an *eve* enhancer fragment fused with the basal *eve* promoter (from −42 bp) and the intact 100 bp untranslated leader and the first 22 codons of the *eve* gene fused with *lac*Z as described [20]. The M32 *eve*- *lac*Z pCaSpeR plasmid contains an additional *Eco*RI site between MSE3 and MSE2. In this case, the *Eco*R1- *Xba*I fragment was first ligated into the vector, and then after transformation and amplification of the product the *Eco*RI- *Eco*RI fragment containing MSE3 was cloned into the RMCE vector after digestion with *Eco*R1. The correct orientation of MSE3 in the RMCE vector was confirmed by DNA sequencing. The pCaSpeR vectors and the RMCE vector were gifts of Stephen Small.

### Site-specific transgenesis

Transgenic lines were established by BestGene Inc. (Chino Hills, CA 91709 U.S.A) using site-specific transgenesis [21] on line A13 from the laboratory of Stephen Small, which contains a landing site in 96F on chromosome III. Surviving flies were crossed to *y w* and progeny were screened for exchange events, scoring for the loss of *y* and gain of *w*. Recombination events were characterized by PCR amplification of the exchange junctions. PCR characterization of recombination events was carried out using the primers land-1 (5′-TCCGTGGGGTTTGAATTAAC-3′, specific to the 5′ end of landing site sequence) and cassette-1 (5′-GGCAGTTAGTTGTTGACTGTG-3′, specific to the 5′ end of transcript sequence in the reporter cassette) and should yield a positive product of approximately 1300 bp to 1600 bp, depending on the length of the regulatory DNA in the cassette.

### *In situ* hybridization

Embryos (1 h–4 h AED) bearing the four fusion genes, M32_ *lox*, M3_2_ *lox*, M23_ *lox*, M2_3_ *lox*, and MSE2 [24] were collected, fixed and stained for *lac*Z mRNA by *in situ* hybridization and for Eve protein by immunostaining as described [8]. MSE2 expression data was obtained from 1511B, one of three MSE2 bearing lines that were gifts of M. Levine. See Figure S1 for a comparison of 1511B and 1511C expression.

### Quantitative expression data

The scanning of fluorescently stained embryos and image segmentation were performed as described [16]. Embryos were classified temporally as belonging to either C13, or one of eight time classes (T1–T8), each about 6.5 minutes long, in cycle 14A (C14A), as described [6]. Background removal was performed as described [22]. Registration was performed by registering to preexisting integrated *eve* data as described [8]. TF expression data for all proteins except Dichaete were that used [8], with the addition of new D-STAT data starting with C13, averaged from at least ten embryos per each time class. The model was fit to ligand data from 35% to 92% AP. Dichaete data were obtained from the t5:26–50 virtual embryo data [74]. Intensity of the gene expression from the middle 10% of dorsoventral position values was quantified by the ImageJ [75] plot profile function and was not registered to Eve pattern. Quantitative expression data for the 1700 construct (1.7 kb proximal *eve* promoter) was previously published [8], and quantitative MSE3 expression data was obtained from M3_2 data by setting expression in stripe 2 to zero.

### Generation and selection of PWMs

PWMs (Table S2) were constructed as follows. We used SELEX to obtain a distribution of nearly optimal binding sites [76] for Bcd, Cad, Hb, Kr, Kni and Gt and Tll as described [77]. We generated a family of PWMs of differing width for each of these TFs by running MEME [78] v.3.0.4 with parameters “-evt 0.001 -dna -nmotifs 10 -minw A -maxw B -nostatus -mod zoops –revcomp” on different selection rounds of the SELEX data, with A equal to 8 and B usually set to 12 unless the results were unsatisfactory, in which case we increased it to values up to 15. From the scientific literature, we also obtained a D-STAT PWM from Dmitri Papasenko (http://line.bioinfolab.net/webgate/help/dxp.htm#D-stat-223), footprint derived PWMs for Tll [79], other footprint factors [80], and bacterial one-hybrid PWMs [81]. We compared these PWMs to each other and those obtained by SELEX as follows. With the threshold set to zero, we discarded all PWMs that failed to detect more than 70% of known footprint sites (Text S1) by extending each site by 5 base pairs of contiguous genomic sequence on each side and considering the highest score of the extended site. From the remaining PWMs, we selected the one that gave the smallest number of false positives when tested against a total of fifteen segments of sequence (20 bp each) from the *eve* transcript which show no peaks on ChIP-Chip assays [82], and unprotected sequence located between known footprint sites. The result, summarized in Table S3, led to the selection of Bcd, Hb, Kr, and Gt sites from our SELEX data, Kni, Dichaete, and Cad sites from bacterial one-hybrid data [81], D-STAT from D. Papasenko, and Tll from a published source [79].

### Identification of new stripe 2 enhancers

The *eve* stripe 2 enhancers from *Drosophila persimilis, mojavensis, grimshawi*, and *willistoni* were identified in the course of this study. To do so, we used a publicly available BLAST tool [83], [84]. We used the *D. melanogaster eve* coding sequence (2R:5866746-5868284) as a query sequence and then scanned 25 kb centered on this region with the two conserved S2E sequences block-A (5′-AATATAACCCAAT-3′) and block-B (5′-TGATTATATCATCATAATAAATGTTT-3′) which bracket the ends of S2E [51]. This provided sequence for S2E's from *mojavensis* and *grimshawi*. In the case of *willistoni*, there is no conserved block-B so we used 1100 bp of sequence 3′ from the conserved block-A. We used 1100 bp because it is approximately the same length as the longest S2E example in our hands, that of *mojavensis* (1089 bp). In the case of *persimilis*, it was not possible to obtain more than 753 bp of sequence 3′ from block-A because the genomic database of this species lacks genomic sequence information beyond this point. We ran the model to predict gene expression from these putative enhancers and the results are shown in Figure 4.

### Computation and optimization

The model equations shown in Figure 2 and Figure 3 were implemented in C. Parameters were determined by minimizing the summed squared difference between the model output and the data, which consisted of 406 observations of RNA level. Optimization was performed using the simulated annealing schedule of Lam [65], [85], [86]. Parameter search spaces were set by explicit search limits for 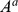, 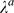, 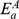,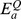, 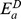, 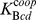, 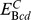, 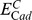 and 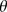 with 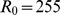 and 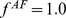 (Figure 2 and Figure 3). Each annealing run required from one to ten days of computation on a single P4 (2.8 GHz) or Xeon (2.6 GHz) processor. Runs were repeated 10 times with different random seeds for each optimization problem. The quality of the runs was judged by its root mean square (rms) score and by visual observation of the expression pattern.

### Implementation of cooperativity and coactivation

The details of our implementation of cooperativity and coactivation are described here. Quenching was implemented as described [8], and Figure S2A of this work], based on published data [38]. We incorporated cooperative binding for Bcd into the model for two reasons. First, there is independent evidence that Bcd binds cooperatively. Second. the model cannot correctly reproduce stripe 2 expression driven by M32 without it [32]. Faced with this observation, we noted that models in which binding affinities 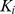 are free parameters could fit this data well when the bcd-4 and bcd-5 (Figure S6) binding sites had identical affinities, even though bcd-4 has a much lower affinity than bcd-5 based on PWM score. This scenario frequently indicates cooperativity [28], and independent experiments have indeed demonstrated pairwise cooperativity between Bcd molecules bound to adjacent sites *in vitro* [30], [31]. Remarkably, the cooperative interaction has a range of at least 41 bp, the center to center distance between the A1 and X1 sites in the *hb* promoter [30]. Given the absence of a well defined upper limit for the range of cooperative interactions of Bcd, we chose a 60 bp range for the studies presented here, although a shorter range did not affect the quality of fit (Table S1, Model 2).

With regard to coactivation, we represented the coactivation range of 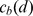 for coactivator 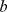 such that the function equals to 1 for 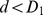 and 0 for 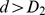, with linear interpolation between these points (Figure S2B). We set 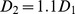 so that only one free parameter is added when coactivation distance is not fixed. Transfection studies on tissue culture cells show that Bcd coactivates Hb [18], so for Bcd we allow 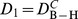 to vary within a range tightly constrained by experimental observations. If 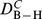 were less than 150 bp, the distance between two closest sites of Bcd bound to MSE2 and Hb bound to MSE3 in the M32 construct, the Hb bound to MSE3 would repress stripe 2 (Figure S2C). If, on the other hand, the distance were longer than 200 bp, a spacer of 160 bp would not suffice to make MSE2 and MSE3 independent in M2_3 (Figure S2D). Training runs gave very constrained values of 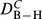 that ranged from 158 to 165 bp (Table S1). In addition to coactivation by Bcd, we permit coactivation of Hb by Cad in the model. In the absence of such coactivation, the model does not permit MSE3 to drive stripe 7 expression. This is unsurprising because Hb sets the anterior border of stripe 3 by repression, and yet stripe 7, driven by MSE3, is located in the interior of the posterior Hb domain. These facts strongly suggest that Hb is coactivated in the region of stripe 7, where Bcd is absent. In contrast to Bcd, there were no independent constraints on the range of the parameter 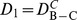 for Cad, so we allowed it to vary from 10 to 200 bp. Training runs gave values between 24 and 70 bp.

### Diffusion limited Arrhenius rate law

The diffusion limited Arrhenius rate law (Figure 3, Equation 10) was derived from a stochastic three state Markov process model, derived from a minimal model of diffusion-limited transcription initiation [87]. We imagine that the system can have the following three states, in which 1) there is no PolII bound to the basal promoter; 2) there is a PolII bound to the basal promoter, but the PolII is stalled; 3) there is a PolII bound to the basal promoter and transcription is initiated, but a new PolII cannot yet bind. Transitions can occur between states 1 and 2 in either direction, but state 3 can only be reached from state 2 and can only change to state 1. Every time the system enters state 3, one new transcript is initiated.

The probabilities 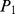, 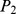, and 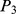 of finding the system in states 1, 2, and 3 respectively are governed by
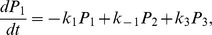


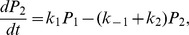


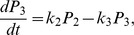
where the 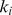 are first order rate constants. We wish to calculate the steady state probabilities 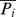 in terms of the kinetic rate constants 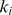. In a steady state the derivatives vanish and we make use of the fact that probabilities add up to one, allowing us to write
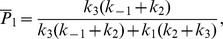


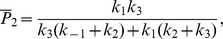


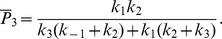


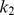
 is the rate-limiting Arrhenius term used in previous non-diffusion limited versions of this model [4], [8], given by
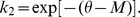
The rate of transcription will be the probability of finding the system in state 3, given by
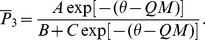
In the absence of detailed kinetic information, we take 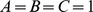 to obtain Equation 10 in Figure 3.

## Supporting Information

Figure S1 Position Effect on Reporter Construct Expression. Segmented expression data with background removed superimposed from multiple embryos bearing P-element transformed or RMCE transformed reporters. The number of embryos used to generate the expression data shown is given in parentheses in each key, and black arrows indicate the maximum expression level found in each construct. (A) Expression of two P-element transformed lines bearing MSE2, 1511B and 1511A [24]. 1511B bears a reporter construct on the second chromosome and 1511C bears the same construct on the third chromosome. (B) Expression of two M32 RMCE transformed M32A and M32B lines bearing the reporter at the same integration site on the second chromosome. The expression levels of M32A and M32B are indistinguishable.
(TIF)

Figure S2Repression and Coactivation Functions. (A) The short range repression function 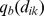. (B) The coactivation function 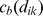. 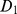 and 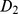 are indicated. Key binding sites used for establishing the coactivation range of Bcd in M32(C) and M2_3 (D) are shown. Bcd and Hb sites are in red and cyan respectively. Some sites are labeled by name. See Figure S6 for a diagram of all sites.(TIF)

Figure S3Training the Model on Four Constructs. (A) The behavior of model 4cs_7 is shown with comparison to expression data, as indicated in the key. The 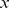-axis is the percentage of A-P position and the 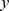-axis is the relative mRNA concentration as described in Figure 1. This model was trained on expression data driven by the four constructs M3_2, M32, M2_3, and M23 only. (B) For comparison, we show the behavior of model 6, trained on seven constructs, compared to training data for the same four constructs shown in (A). The behavior of model 6 compared to its full training set is shown in Figure 4A1-7 and Figure S4. Note that model 4cs_7 fits the expression data driven by M32 better than model 6. Comparative rms scores are shown at the top. The full set of parameters for each model is given in Table S1.(TIF)

Figure S4Training the Model on Seven Constructs. Model output is represented by the red solid lines, while the observed expression data is represented by the black dashed lines, as shown in the key. The behavior of models 1, 2, 6, and 7 are shown as indicated in the leftmost column, which also gives each model's rms score. Parameter sets for these four models are given in Table S1. The 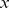-axis is the percentage of A-P position and the 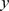-axis is the relative mRNA concentration as described in Figure 1. Note that the concentration scale for model 7 differs from the other two rows. The data is rescaled by the factor 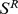, a free parameter for position effect, for the P-transformed constructs 1700, MSE2, and MSE3 (Table S1).(TIF)

Figure S5 Incorrect Predictions. Incorrect predictions of gene expression driven by DNA sequences that were not used for training. The sequences used are fully described in Table S5. Black lines are predicted RNA expression and colored lines are quantitative protein profiles of the corresponding endogenous loci. The scale of relative fluorescence levels for RNA is shown at the left of graphs, that for proteins on the right. All protein patterns are taken from the FlyEx database (http://urchin.spbcas.ru/flyex) [7]. All predictions in this Figure were made using the model 6 parameters (Table S1). (A–B) Predictions for the *eve* stripe 5 (A) and 4/6 enhancers (B). Correct predictions of these enhancers from model 2 are shown in Figure 4D1-2. (C–L) Predicted expression driven by enhancers from the genes *hb* (C), *kni* (D–E), *gt* (F–G), *run* (H), and *h* (I–L). (M–N) Predictions for expression driven by large 5′ (M) and 3′ (N) *eve* regulatory DNAs that contain multiple enhancers. Correct predictions for these DNA segments from models 7 and 1 respectively are shown in Figure 4G1-2.
(TIF)

Figure S6 All Binding Sites Used in Model 6. Every binding site used in model 6 is shown. Of these, all footprint sites of the four TFs Bcd, Hb, Kr, Gt are numbered as the same way as in the original papers [17], [18]. (A) 5′ upstream of *eve*. (B) M3_2 (C) M32 (D) M2_3 (E) M23. Key rearrangements of binding sites are indicated by black arrows. bcd-(−1) is a computationally identified site named in this work. bcd-* is evident on footprints [24], but was not named.
(TIF)

Figure S7Regulatory Analysis of M2_3 and M23. (A) Zones V and VI, the areas where expression changes occur between M23 and M2_3. (B–C) Distribution of activation energy barrier changes at single binding site resolution for M2_3 and M23 as a function of A-P position on the embryo and number of basepairs 5′ to their transcription start site. In (B) and (C) the positions of MSE2 and MSE3 are schematically shown at the top. 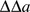 for each activator binding site is shown in the central panel according to the key in Figure 5B and the summed activation 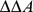 in the right hand bar. All footprints sites for Bcd, D-STAT, Hb, Kr and Gt are shown at the top of panels (B) and (C) except for the Kr-2 site in the spacer (Figure S6D), which is very close to the 3′ Bcd site in the spacer. Computationally identified Cad binding sites in MSE3 and Bcd sites in the spacer are also shown. The black arrows in (B) and (C) indicate two Hb sites potentially subject to coactivation by Bcd. The red arrow indicates which of these sites is in fact subject to coactivation in a given construct. Circled areas highlight major changes in 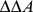 between M2_3 and M23, and the white arrows indicate which binding sites cause the changes seen in the circled areas. The distributions of TFs and further information about the diagrams in (B) and (C) are given in Figure 5D and its legend.(TIF)

Table S1Parameters of 5 Models. These parameters are inferred from the observed expression patterns by fitting transcription models to quantitative data. Daggers indicate parameters held fixed during the training process. 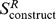 is the positional effect scale factor for each reporter construct. 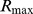 is the maximum rate of transcription. 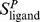 is the scale factor for protein concentration. Other parameters are described in the main text.(PDF)

Table S2 Alignment Matrices Used in the Model. For each PWM, the left most column indicates DNA bases. The remaining columns show the number of observed bases at each position in the binding site.
(PDF)

Table S3 Comparison Between PWMs. For each TF, the top row is the recovery rate of footprint sites and the bottom row is the rate of false positives.
(PDF)

Table S4 Regulatory Sequences Used for Predictions. All DNA sequences used in this work are listed here. Index indicates the figure panel where the results of the prediction are shown. Name indicates the sequence designator used in that panel. DNA source gives the source of the sequence itself, and Reference where it was first described. We give the genomic position if known. Asterisks in the second column indicate that there were small differences between the regulatory sequences we utilized and the corresponding sequences available in FlyBase (http://www.flybase.org). The REDfly database is at http://redfly.ccr.buffalo.edu. Full sequences first identified in this work are listed in Text S2.
(PDF)

Table S5 *Drosophila* and Sepsid Species Abbreviations. For each full species name, the first word indicates the genus and the second word indicates species.
(PDF)

Text S1 Comparison of Recovery Rate and False Positive Rate Between PWMs. The first matrix in each table is the PWM used in this work. The remaining matrices are used for comparison.
(TXT)

Text S2 Full Sequences First Identified in This Work. *eve* stripe 2 enhancer sequences identified in this work are listed in FASTA format. The abbreviation for each species name is shown in the sequence name.
(TXT)
