# Supplementary figures and images for: Rearrangements of 2.5 Kilobases of Noncoding DNA from the Drosophila even-skipped Locus Define Predictive Rules of Genomic cis-Regulatory Logic
Source: PLoS Genet. 2013 Feb 28;9(2):e1003243. doi: 10.1371/journal.pgen.1003243 (PMC3585115; doi:10.1371/journal.pgen.1003243)

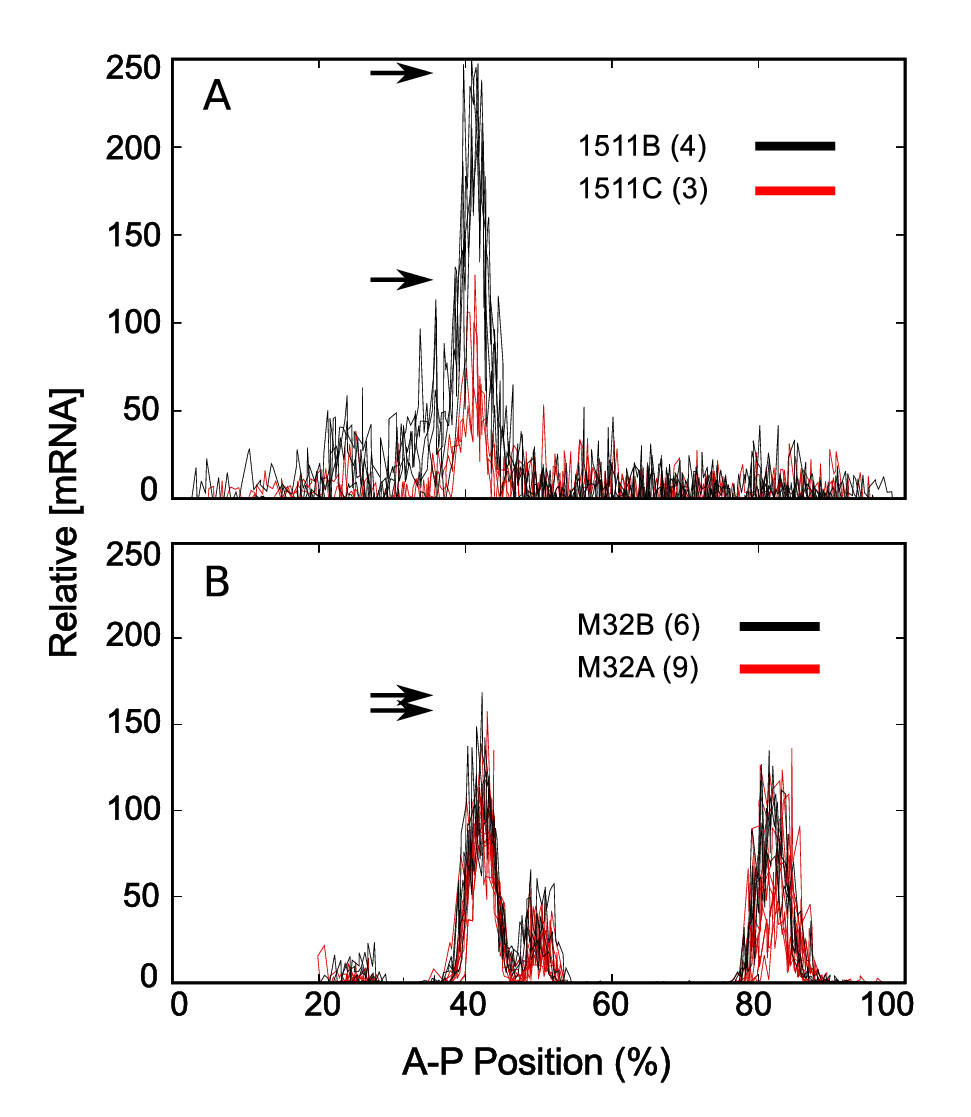

Supplement: Figure S1 — Position Effect on Reporter Construct Expression. Segmented expression data with background removed superimposed from multiple embryos bearing P-element transformed or RMCE transformed reporters. The number of embryos used to generate the expression data shown is given in parentheses in each key, and black arrows indicate the maximum expression level found in each construct. (A) Expression of two P-element transformed lines bearing MSE2, 1511B and 1511A [24]. 1511B bears a reporter construct on the second chromosome and 1511C bears the same construct on the third chromosome. (B) Expression of two M32 RMCE transformed M32A and M32B lines bearing the reporter at the same integration site on the second chromosome. The expression levels of M32A and M32B are indistinguishable. (TIF) [file pgen.1003243.s001.tif]

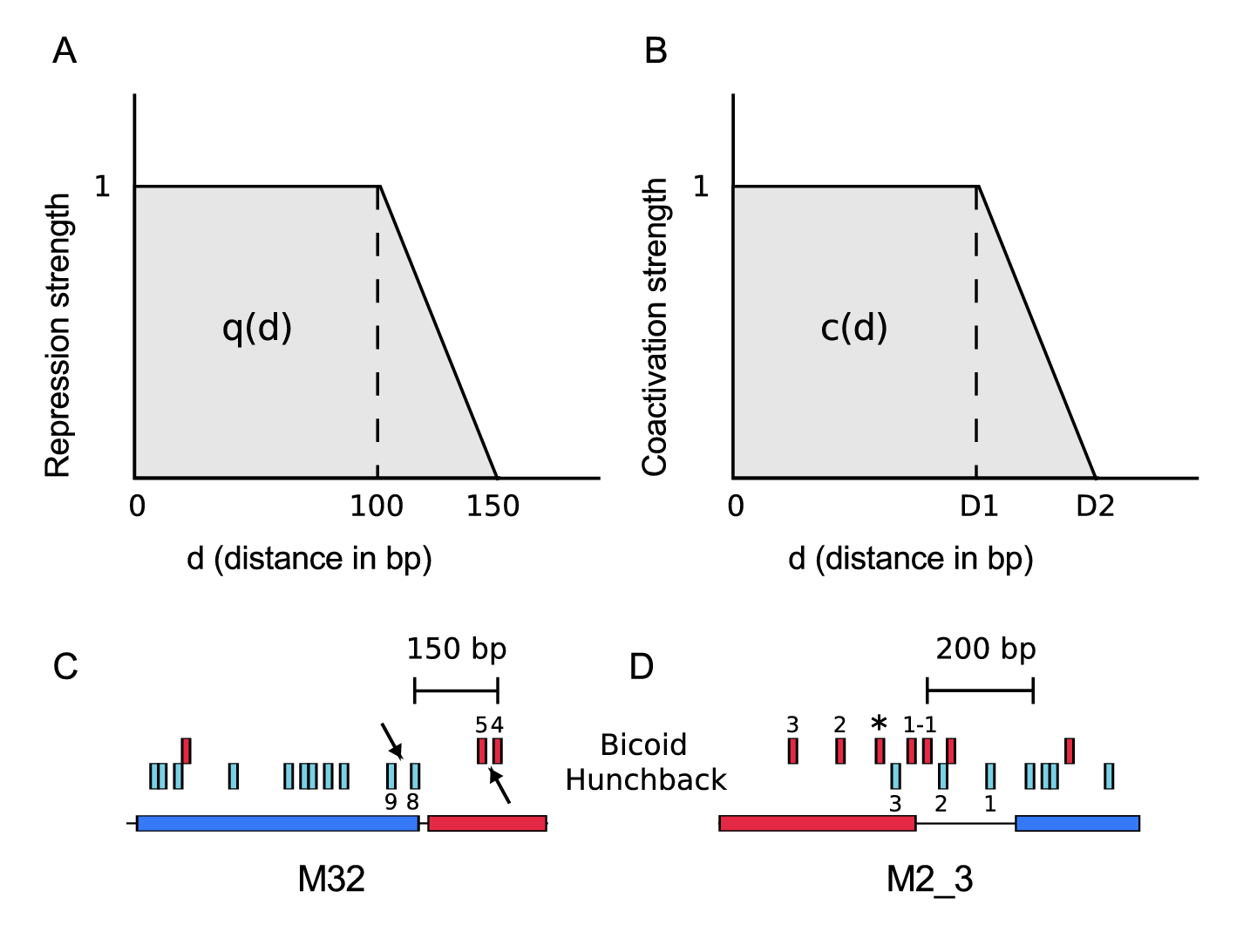

Supplement: Figure S2 — Repression and Coactivation Functions. (A) The short range repression function . (B) The coactivation function . and are indicated. Key binding sites used for establishing the coactivation range of Bcd in M32(C) and M2_3 (D) are shown. Bcd and Hb sites are in red and cyan respectively. Some sites are labeled by name. See Figure S6 for a diagram of all sites. (TIF) [file pgen.1003243.s002.tif]

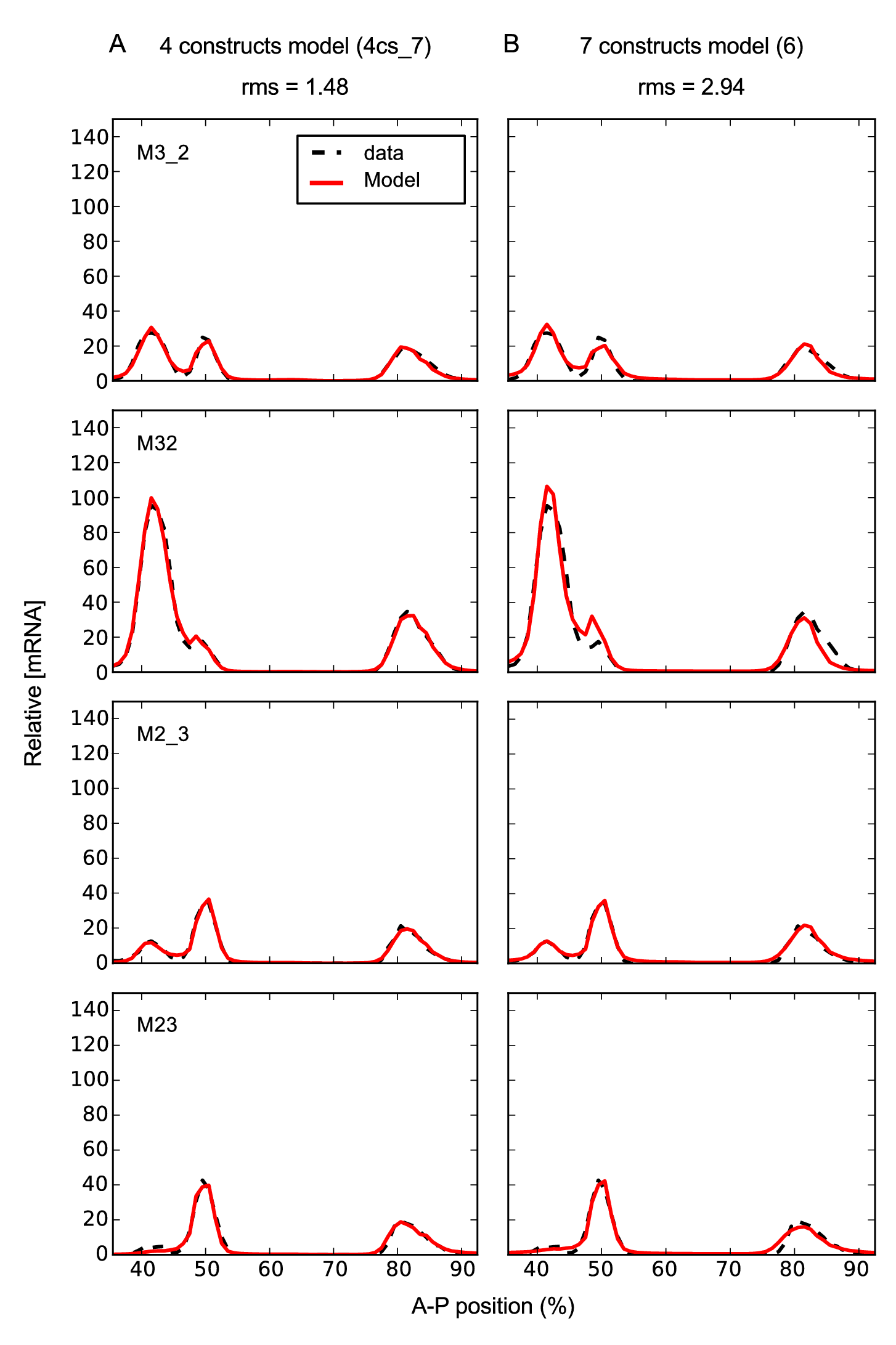

Supplement: Figure S3 — Training the Model on Four Constructs. (A) The behavior of model 4cs_7 is shown with comparison to expression data, as indicated in the key. The -axis is the percentage of A-P position and the -axis is the relative mRNA concentration as described in Figure 1. This model was trained on expression data driven by the four constructs M3_2, M32, M2_3, and M23 only. (B) For comparison, we show the behavior of model 6, trained on seven constructs, compared to training data for the same four constructs shown in (A). The behavior of model 6 compared to its full training set is shown in Figure 4A1-7 and Figure S4. Note that model 4cs_7 fits the expression data driven by M32 better than model 6. Comparative rms scores are shown at the top. The full set of parameters for each model is given in Table S1. (TIF) [file pgen.1003243.s003.tif]

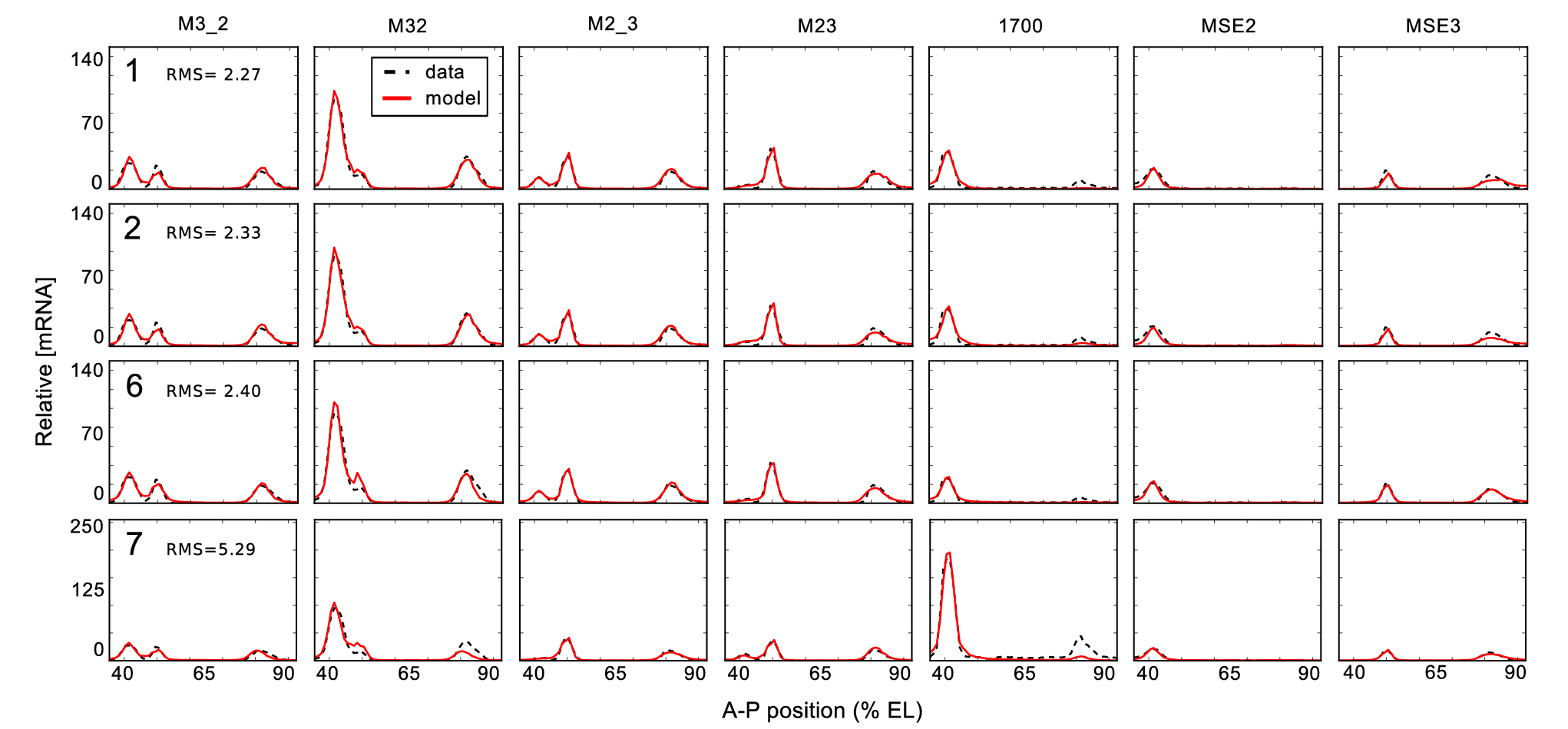

Supplement: Figure S4 — Training the Model on Seven Constructs. Model output is represented by the red solid lines, while the observed expression data is represented by the black dashed lines, as shown in the key. The behavior of models 1, 2, 6, and 7 are shown as indicated in the leftmost column, which also gives each model's rms score. Parameter sets for these four models are given in Table S1. The -axis is the percentage of A-P position and the -axis is the relative mRNA concentration as described in Figure 1. Note that the concentration scale for model 7 differs from the other two rows. The data is rescaled by the factor , a free parameter for position effect, for the P-transformed constructs 1700, MSE2, and MSE3 (Table S1). (TIF) [file pgen.1003243.s004.tif]

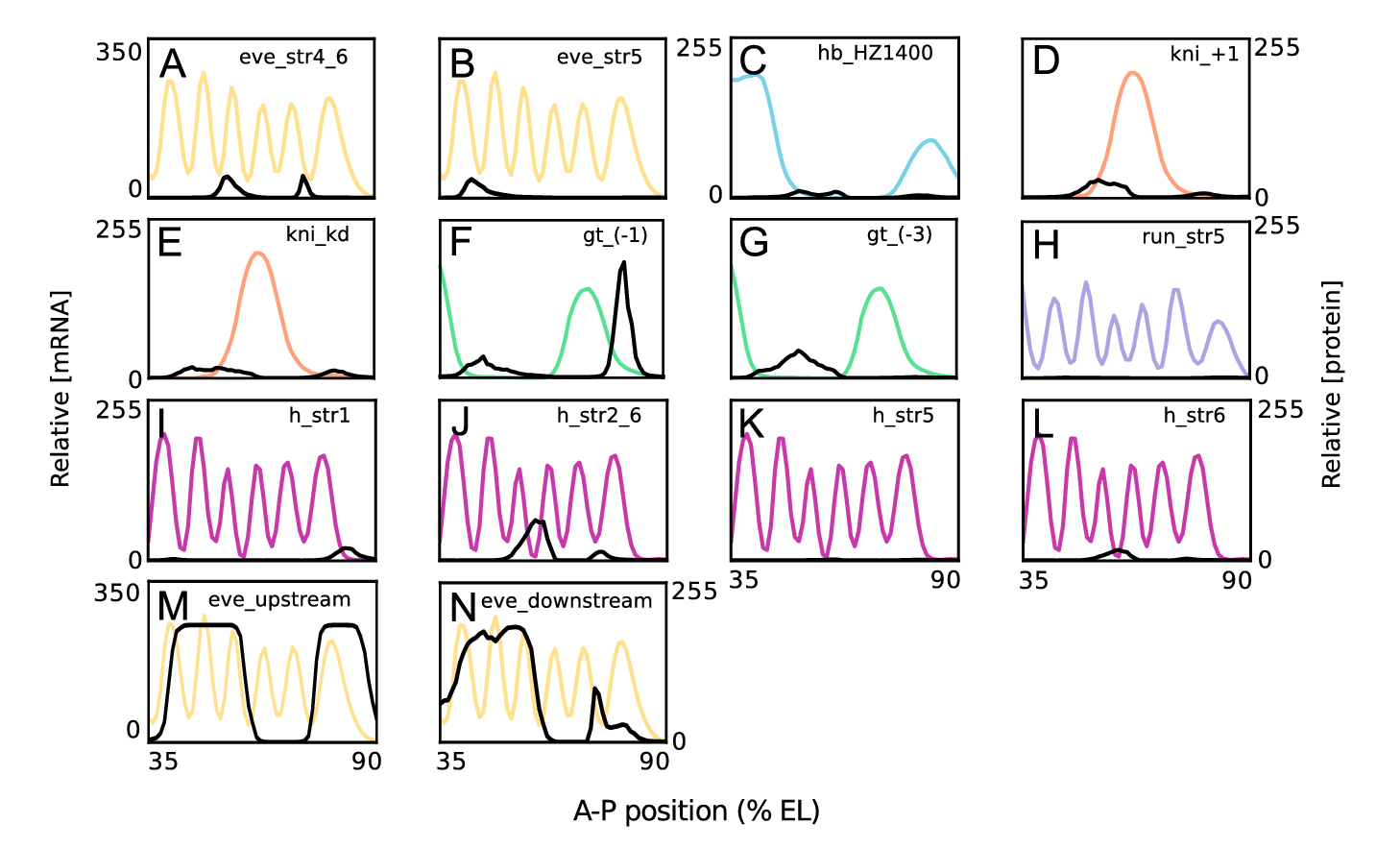

Supplement: Figure S5 — Incorrect Predictions. Incorrect predictions of gene expression driven by DNA sequences that were not used for training. The sequences used are fully described in Table S5. Black lines are predicted RNA expression and colored lines are quantitative protein profiles of the corresponding endogenous loci. The scale of relative fluorescence levels for RNA is shown at the left of graphs, that for proteins on the right. All protein patterns are taken from the FlyEx database (http://urchin.spbcas.ru/flyex) [7]. All predictions in this Figure were made using the model 6 parameters (Table S1). (A–B) Predictions for the eve stripe 5 (A) and 4/6 enhancers (B). Correct predictions of these enhancers from model 2 are shown in Figure 4D1-2. (C–L) Predicted expression driven by enhancers from the genes hb (C), kni (D–E), gt (F–G), run (H), and h (I–L). (M–N) Predictions for expression driven by large 5′ (M) and 3′ (N) eve regulatory DNAs that contain multiple enhancers. Correct predictions for these DNA segments from models 7 and 1 respectively are shown in Figure 4G1-2. (TIF) [file pgen.1003243.s005.tif]

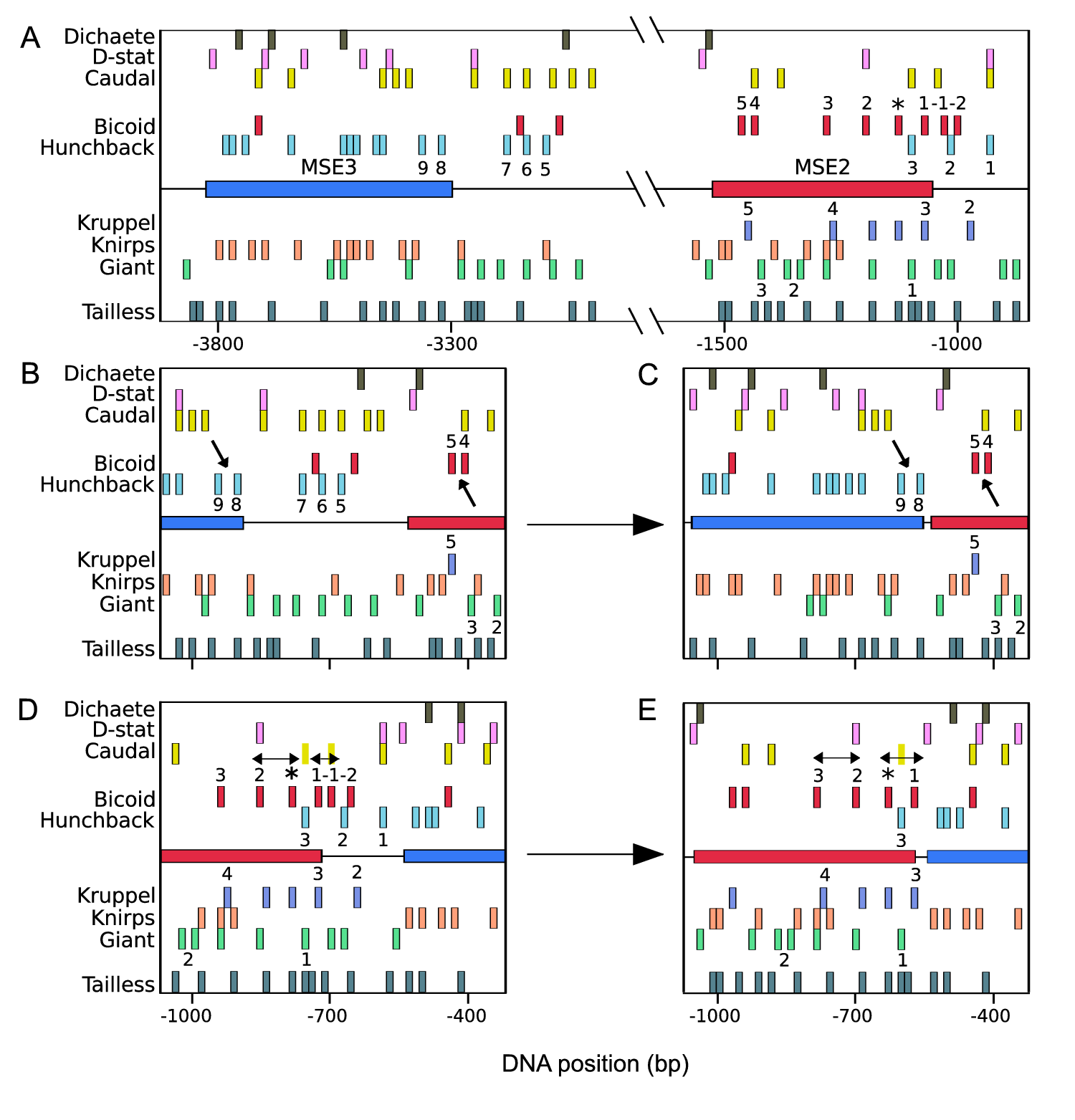

Supplement: Figure S6 — All Binding Sites Used in Model 6. Every binding site used in model 6 is shown. Of these, all footprint sites of the four TFs Bcd, Hb, Kr, Gt are numbered as the same way as in the original papers [17], [18]. (A) 5′ upstream of eve. (B) M3_2 (C) M32 (D) M2_3 (E) M23. Key rearrangements of binding sites are indicated by black arrows. bcd-(−1) is a computationally identified site named in this work. bcd-* is evident on footprints [24], but was not named. (TIF) [file pgen.1003243.s006.tif]

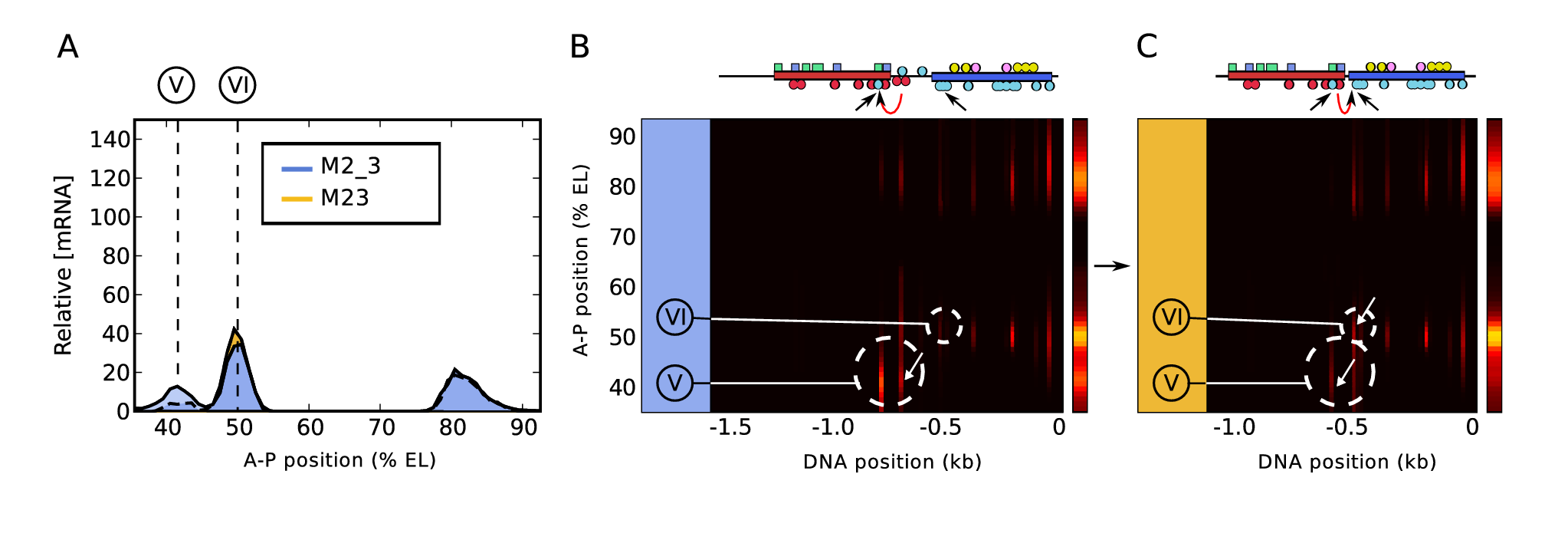

Supplement: Figure S7 — Regulatory Analysis of M2_3 and M23. (A) Zones V and VI, the areas where expression changes occur between M23 and M2_3. (B–C) Distribution of activation energy barrier changes at single binding site resolution for M2_3 and M23 as a function of A-P position on the embryo and number of basepairs 5′ to their transcription start site. In (B) and (C) the positions of MSE2 and MSE3 are schematically shown at the top. for each activator binding site is shown in the central panel according to the key in Figure 5B and the summed activation in the right hand bar. All footprints sites for Bcd, D-STAT, Hb, Kr and Gt are shown at the top of panels (B) and (C) except for the Kr-2 site in the spacer (Figure S6D), which is very close to the 3′ Bcd site in the spacer. Computationally identified Cad binding sites in MSE3 and Bcd sites in the spacer are also shown. The black arrows in (B) and (C) indicate two Hb sites potentially subject to coactivation by Bcd. The red arrow indicates which of these sites is in fact subject to coactivation in a given construct. Circled areas highlight major changes in between M2_3 and M23, and the white arrows indicate which binding sites cause the changes seen in the circled areas. The distributions of TFs and further information about the diagrams in (B) and (C) are given in Figure 5D and its legend. (TIF) [file pgen.1003243.s007.tif]
