# Supplementary material for: Rearrangements of 2.5 Kilobases of Noncoding DNA from the Drosophila even-skipped Locus Define Predictive Rules of Genomic cis-Regulatory Logic
Source: PLoS Genet. 2013 Feb 28;9(2):e1003243. doi: 10.1371/journal.pgen.1003243 (PMC3585115; doi:10.1371/journal.pgen.1003243)
Supplement: Table S1 — Parameters of 5 Models. These parameters are inferred from the observed expression patterns by fitting transcription models to quantitative data. Daggers indicate parameters held fixed during the training process. is the positional effect scale factor for each reporter construct. is the maximum rate of transcription. is the scale factor for protein concentration. Other parameters are described in the main text. (PDF) [file pgen.1003243.s008.pdf]

Table S1. Parameters of 5 Models

Parameter	Run number					Parameter	Run number				
	1	2	6	7	4cs_7		1	2	6	7	4cs_7
RMS	2.27	2.33	2.40	5.29	1.48	A^P_{Bcd}	1.36	0.69	0.03	0.08	3.99
$S^R_{\mathrm{M3\_2}}$†	1	1	1	1	1	A^P_{Cad}	0.02	0.01	0.05	0.03	0.05
S^R_{M32}†	1	1	1	1	1	$A^P_{\mathrm{D-STAT}}$	3.98	3.98	1.09	3.99	3.98
$S^R_{\mathrm{M2\_3}}$†	1	1	1	1	1	A^P_{Dichaete}	3.85	0.41	3.89	0.18	0.09
S^R_{M23}†	1	1	1	1	1	A^P_{Hb}	0.82	2.73	2.72	0.04	0.09
$S^R_{\mathrm{1.7kb}}$	0.3	0.3	0.2	1.41	N/A	A^P_{Kr}	0.07	0.13	0.03	2.95	0.1
S^R_{MSE2}	0.2	0.2	0.2	0.2	N/A	A^P_{Kni}	2.58	3.99	2.23	0.27	2.8
S^R_{MSE3}	0.8	0.8	0.8	0.8	N/A	A^P_{Gt}	3.99	2.07	2.53	0.04	0.02
R_{max}†	255	255	255	255	255	A^P_{Tll}	2.88	3.96	0.02	1.95	0.004
Θ	6.7	6.4	6.1	5.96	10.6	λ_{Bcd}	1.53	1.99	4.99	2.16	1.68
$E^C_{\mathrm{B-H}}$	0.34	0.44	0.21	0.35	0.35	λ_{Cad}	4.98	4.98	4.97	3.18	4.99
$E^C_{\mathrm{C-H}}$	0.66	0.99	0.33	0.88	0.88	$\lambda_{\mathrm{D-STAT}}$	1.62	1.98	2.58	0.69	0.89
$K^{\mathrm{coop}}_{\mathrm{B-B}}$	52	982	189	127	86	$\lambda_{\mathrm{Dichaete}}$	0.91	2.33	1.98	4.54	3.49
$D^C_{\mathrm{B-H}}$	165	161	158	150	150	λ_{Hb}	1.93	1.5	1.83	4.99	4.25
$D^C_{\mathrm{C-H}}$	57	58	70	22	28	λ_{Kr}	3.08	2.31	4.04	0.98	4.99
$D^{\mathrm{coop}}_{\mathrm{B-B}}$†	60	20	60	60	60	λ_{Kni}	1.6	1.17	2.48	1.56	1.82
E^A_{Bcd}	0.5	0.5	0.06	0.0001	0.001	λ_{Gt}	1.25	1.47	1.71	4.99	4.63
E^A_{Cad}	0.0001	0.0001	0.0001	0.0001	0.39	λ_{Tll}	0.87	1.26	4.98	0.96	4.99
$E^A_{\mathrm{D-STAT}}$	19.9	19.9	0.0001	19.9	16.6	T_{Bcd}†	1.71	1.71	1.71	1.71	1.71
E^A_{Dichaete}	0.0001	0.0001	0.0001	0.45	0.004	T_{Cad}	2.53	2.22	3.06	2.06	3.0
E^A_{Hb}	14.4	13.5	20.41	29.9	19.1	$T_{\mathrm{D-STAT}}$	2.21	2.19	2.83	3.63	2.83
E^Q_{Hb}	0.99	0.99	0.99	0.99	0.99	T_{Dichaete}	2.22	4.92	4.79	2.96	2.08
E^Q_{Kr}	0.99	0.99	0.9	0.99	0.51	T_{Hb}†	0.63	0.63	0.63	0.63	0.63
E^Q_{Kni}	0.54	0.75	0.06	0.99	0.26	T_{Kr}	0.009	0.02	2.11	0.07	2.06
E^Q_{Gt}	0.75	0.43	0.72	0.74	0.99	T_{Kni}	2.2	2.48	2.23	4.85	2.46
E^Q_{Tll}	0.99	0.14	0.99	0.99	0.81	T_{Gt}	0.6	0.59	0.59	0.50	0.71
E^D_{Hb}	0.53	0.31	0.32	0.58	0.37	T_{Tll}	1.83	1.82	1.97	1.97	1.97
E^D_{Kr}	0.99	0.73	0.99	0.99	0.6						
E^D_{Kni}	0.24	0.14	0.12	0.99	0.05						
E^D_{Gt}	0.87	0.99	0.17	0.99	0.51						
E^D_{Tll}	0.0001	0.0001	0.0002	0.0001	0.98						
D^Q_{all}†	100	100	100	100	100						
D^D_{all}†	100	100	100	100	100						

These parameters are inferred from the observed expression patterns by fitting transcription models to quantitative data. Daggers indicate parameters held fixed during the training process. $S^R_{\mathrm{construct}}$ is the positional effect scale factor for each reporter construct. R_{max} is the maximum rate of transcription. S^P_{ligand} is the scale factor for protein concentration. Other parameters are described in the main text.
