# Supplementary material for: Rearrangements of 2.5 Kilobases of Noncoding DNA from the Drosophila even-skipped Locus Define Predictive Rules of Genomic cis-Regulatory Logic
Source: PLoS Genet. 2013 Feb 28;9(2):e1003243. doi: 10.1371/journal.pgen.1003243 (PMC3585115; doi:10.1371/journal.pgen.1003243)
Supplement: Table S2 — Alignment Matrices Used in the Model. For each PWM, the left most column indicates DNA bases. The remaining columns show the number of observed bases at each position in the binding site. (PDF) [file pgen.1003243.s009.pdf]

Table S2. Alignment Matrices Used in the Model

Bicoid

A	83	74	108	48	6	381	379	5	0	6	72	61	65	68
C	114	159	127	149	1	0	0	0	383	340	136	174	166	158
G	106	72	114	11	0	2	4	4	0	3	132	60	52	49
T	80	78	34	175	376	0	0	374	0	34	43	88	100	108

Caudal

A	9	12	3	4	12	38	0	4	22	1
C	10	6	3	0	0	0	0	0	0	8
G	4	4	3	0	2	0	0	7	15	10
T	11	16	29	34	24	0	38	27	1	1

D-STAT

A	1	1	2	1	1	5	3	0	24	28	27	5
C	0	0	1	27	20	16	3	2	3	0	1	8
G	0	1	0	1	6	8	22	27	1	1	0	6
T	29	28	27	1	3	1	2	1	2	1	2	1

Dichaete

A	1	0	0	20	0	0	2	0	1	4	6
C	8	25	17	0	0	0	0	0	2	10	1
G	7	0	0	0	0	3	27	0	4	6	1
T	13	4	12	9	29	26	0	29	22	9	21

Hunchback

A	53	2	0	2	0	0	0	281	31	20
C	6	6	2	0	2	3	2	0	43	100
G	224	3	0	0	0	0	0	3	78	109
T	7	279	288	288	288	287	288	6	138	61

Kruppel

A	17	187	158	0	1	0	8	0	2	44
C	73	5	39	194	194	197	22	2	34	109
G	6	0	0	1	0	0	6	0	2	15
T	101	5	0	2	2	0	161	195	159	29

Knirps

A	19	25	16	5	0	21	0	17	1	0	25	5
C	1	1	0	9	4	0	0	0	3	26	0	12
G	2	0	0	6	1	5	26	8	18	0	1	7
T	4	0	10	6	21	0	0	1	4	0	0	2

Giant

A	86	12	776	8	83	0	1020	1106	15
C	62	108	25	762	19	556	88	0	378
G	19	359	275	65	996	0	1	0	85
T	942	630	33	274	11	553	0	3	631

Tailless

A	12	1	1	5	2	11	1	0	0
C	8	2	2	1	3	1	17	2	3
G	0	2	1	0	15	5	0	1	2
T	0	15	16	14	0	3	2	17	15

For each PWM, the left most column indicates DNA bases. The remaining columns show the number of observed bases at each position in the binding site.
