# Supplementary material for: Rearrangements of 2.5 Kilobases of Noncoding DNA from the Drosophila even-skipped Locus Define Predictive Rules of Genomic cis-Regulatory Logic
Source: PLoS Genet. 2013 Feb 28;9(2):e1003243. doi: 10.1371/journal.pgen.1003243 (PMC3585115; doi:10.1371/journal.pgen.1003243)
Supplement: Table S3 — Comparison Between PWMs. For each TF, the top row is the recovery rate of footprint sites and the bottom row is the rate of false positives. (PDF) [file pgen.1003243.s010.pdf]

Table S3. Comparison Between PWMs

	**This work**	**Berman *et al.* [1]**	**Segal *et al.* [2]**
**Bcd**	79% (38/48)	100% (48/48)	58% (28/48)
	8% (3/35)	42% (15/35)	14% (5/35)
**Cad**	76% (10/13)	100% (13/13)	69% (9/13)
	15% (4/26)	100% (26/26)	19% (5/26)
**D-STAT**	100% (3/3)	N/A	0% (0/3)
	17% (3/17)	N/A	17%(3/17)
**Dichaete**	100% (4/4)	N/A	N/A
	22% (4/18)	N/A	N/A
**Hb**	87% (90/103)	98% (101/103)	86% (89/103)
	3% (1/26)	19% (5/26)	0% (0/26)
**Kr**	75% (34/45)	86% (39/45)	86% (39/45)
	6% (1/15)	26% (4/15)	40% (6/15)
**Kni**	75% (25/33)	84% (28/33)	42% (14/33)
	21% (9/42)	59% (25/42)	28% (12/42)
**Gt**	75% (6/8)	N/A	62% (5/8)
	6% (1/15)	N/A	20% (3/15)
**Tll**	94% (35/37)	N/A	70% (26/37)
	63% (23/26)	N/A	52% (19/36)

For each TF, the top row is the recovery rate of footprint sites and the bottom row is the rate of false positives.

References

1. Berman BP, Nibu Y, Pfeiffer BD, Tomancak P, Celniker SE, et al. (2002) Exploiting transcription factor binding site clustering to identify cis-regulatory modules involved in pattern formation in the *Drosophila* genome. Proceedings of the National Academy of Sciences USA 99: 757-762.

2. Segal E, Raveh-Sadka T, Schroeder M, Unnerstall U, Gaul U (2008) Predicting expression patterns from regulatory sequence in *Drosophila* segmentation. Nature 451: 535-540.
