# Supplementary material for: Rearrangements of 2.5 Kilobases of Noncoding DNA from the Drosophila even-skipped Locus Define Predictive Rules of Genomic cis-Regulatory Logic
Source: PLoS Genet. 2013 Feb 28;9(2):e1003243. doi: 10.1371/journal.pgen.1003243 (PMC3585115; doi:10.1371/journal.pgen.1003243)
Supplement: Table S4 — Regulatory Sequences Used for Predictions. All DNA sequences used in this work are listed here. Index indicates the figure panel where the results of the prediction are shown. Name indicates the sequence designator used in that panel. DNA source gives the source of the sequence itself, and Reference where it was first described. We give the genomic position if known. Asterisks in the second column indicate that there were small differences between the regulatory sequences we utilized and the corresponding sequences available in FlyBase (http://www.flybase.org). The REDfly database is at http://redfly.ccr.buffalo.edu. Full sequences first identified in this work are listed in Text S2. (PDF) [file pgen.1003243.s011.pdf]

Table S4. Regulatory Sequences Used for Predictions

Index	Name	Length (bp)	DNA source	Reference	Genomic position (bp)
4B1	eve_MSE2Mbcd1	489	[1]	[1]	
4B2	eve_MSE2Mbcd3	489	[1]	[1]	
4B3	eve_MSE3M2dsts	502	[2]	[2]	
4B4	eve_M32_MKr345	1016	[3]	[3]	
4B5	eve_M32_Mbcd1	1016	[3]	[3]	
4C1	S2E(yak)*	844	[4]	[4]	C2L:18492244,18493087
4C2	S2E(pse)*	1027	[4]	[4]	C3:10905710,10906728
4C3	S2E(ere)*	849	[4]	[4]	S4929:8503125,8503973
4C4	S2E(ore)	905	Text S2	This work	
4C5	S2E(tei)	882	Text S2	This work	
4C6	S2E(tak)	742	Text S2	This work	
4C7	S2E(mau)	797	Text S2	This work	
4C8	S2E(sec)*	788	Text S2	This work	S359:11527,12271
4C9	S2E(per)	753	Text S2	This work	S4:6229414,6230166
4C10	S2E(sim)*	798	[4]	[4]	C2R:4497397,4498185
4C11	S2E(ana)*	816	Text S2	This work	S13266:15364458,15365264
4C12	S2E(vir)*	973	Text S2	This work	S12875:1336908,1337886
4C13	S2E(pic)	1036	[5]	[5]	
4C14	S2E(gri)	1065	Text S2	This work	S15245:9655365,9656429
4C15	S2E(moj)	1089	Text S2	This work	S6496:4429248,4430336
4C16	S2E(wil)	1100	Text S2	This work	S180700:33743,34842
4D1	MSE5	804	Redfly	[6]	C2R:5498538,5499341
4D2	MSE4_6	800	Redfly	[6]	C2R:5495712,5496511
4D3	M3_S2(p1-m2)	1544	[7]	[7]	
4D4	M3_S2(m1-p2)	1433	[7]	[7]	
4E1	S2E(cyn)	1939	[8]	[8]	
4E2	S2E(put)	1698	[8]	[8]	
4E3	S2E(sup)	1791	[8]	[8]	
4E4	S2E(dsp)	2437	[8]	[8]	
4E5	S2E(min)	1579	[8]	[8]	
4E6	S2E(pun)	2034	[8]	[8]	
4E7	S37E(cyn)	2044	[8]	[8]	
4E8	S37E(put)	1682	[8]	[8]	
4E9	S37E(sup)	1887	[8]	[8]	
4E10	S37E(dsp)	1540	[8]	[8]	
4E11	S37E(min)	1575	[8]	[8]	
4E12	S37E(pun)	2120	[8]	[8]	
4F1	hb_pThb1_hbp	298	Redfly	[9]	C3R:4520323,4520620
4F2	Kr_CD1_hsp70p	1159	Redfly	[10]	C2R:20730219,20731377
4F3	run_str1_7	1611	Redfly	[11]	CX:20490688,20492298
4F4	run_str3_7	2404	Redfly	[11]	CX:20493864,20496267
4F5	h_str3_4	1745	Redfly	[12]	C3L:8637477,8639221
4G1	eve_ups	3942	FlyBase	[13]	C2R:5487187,5491128
4G2	eve_downs	3500	FlyBase	[6]	C2R:5496129, 5499628

Index	Name	Length (bp)	DNA source	Reference	Genomic position (bp)
S5C	hbHZ1400_hsp70p	1421	Redfly	[9]	C3R:4526522,4527942
S5D	kni_+1	1479	Redfly	[14]	C3L:20533736,20629274
S5E	kni_kd	875	Redfly	[15]	C3L:20630383,20631257
S5F	gt_(-1)	1239	Redfly	[14]	CX:2285171,2286409
S5G	gt_(-3)	1209	Redfly	[14]	CX:2286417,2287625
S5H	run_5	1340	Redfly	[11]	CX:20492298,20493637
S5I	h_str1	876	Redfly	[16]	C3L:8644872,8645747
S5J	h_str2_6	1081	Redfly	[12]	C3L:8640258,8641338
S5K	h_str5	564	Redfly	[17]	C3L:8644027,8644590
S5L	h_str6	547	Redfly	[18]	C3L:8640797,8641343

All DNA sequences used in this work are listed here. Index indicates the figure panel where the results of the prediction are shown. Name indicates the sequence designator used in that panel. DNA source gives the source of the sequence itself, and Reference where it was first described. We give the genomic position if known. Asterisks in the second column indicate that there were small differences between the regulatory sequences we utilized and the corresponding sequences available in FlyBase (http://www.flybase.org). The REDfly database is at http://redfly.ccr.buffalo.edu. Full sequences first identified in this work are listed in Text S2.

References

1. Small S, Blair A, Levine M (1992) Regulation of *even-skipped* stripe 2 in the *Drosophila* embryo. The EMBO Journal 11: 4047-4057.

2. Yan R, Small S, Desplan C, Dearolf CR, Jr JED (1996) Identification of a *stat* gene that functions in *Drosophila* development. Cell 84: 421-430.

3. Small S, Arnosti DN, Levine M (1993) Spacing ensures autonomous expression of different stripe enhancers in the *even-skipped* promoter. Development 119: 767-772.

4. Ludwig MZ, Kreitman M (1995) Evolutionary dynamics of the enhancer region of *even-skipped* in *Drosophila*. Molecular Biology and Evolution 12: 1002-1011.

5. Sackerson C (1995) Patterns of conservation and divergence at the *even-skipped* locus of *Drosophila*. Mechanisms of Development 51: 199-215.

6. Fujioka M, Emi-Sarker Y, Yusibova GL, Goto T, Jaynes JB (1999) Analysis of an *even-skipped* rescue transgene reveals both composite and discrete neuronal and early blastoderm enhancers, and multi-stripe positioning by gap gene repressor gradients. Development 126: 2527-2538.

7. Ludwig MZ, Bergman CM, Patel NH, Kreitman M (2000) Evidence for stabilizing selection in a eukaryotic enhancer element. Nature 403: 564-567.

8. Hare EE, Peterson BK, Iyer VN, Meier R, Eisen MB (2008) Sepsid *even-skipped* enhancers are functionally conserved in *Drosopila* despite lack of sequence conservation. PLoS Genetics 4: e1000106.

9. Margolis JS, Borowsky ML, Steingrimsson E, Shim CW, Lengyel JA, et al. (1995) Posterior stripe expression of *hunchback* is driven from two promoters by a common enhancer element. Development 121: 3067-3077.

10. Hoch M, Schröder C, Seifert E, Jäckle H (1990) *Cis*-acting control elements for *Krüppel* expression in the *Drosophila* embryo. The EMBO Journal 9: 2587-2595.

11. Klingler M, Soong J, Butler B, Gergen JP (1996) Disperse versus compact elements for the regulation of *runt* stripes in *Drosophila*. Developmental Biology 177: 73-84.

12. Howard KR, Struhl G (1990) Decoding positional information: regulation of the pair-rule gene *hairy*. Development 110: 1223-1231.

13. Stanojevic D, Small S, Levine M (1991) Regulation of a segmentation stripe by overlapping activators and repressors in the *Drosophila* embryo. Science 254: 1385-1387.

14. Schroeder MD, Pearce M, Fak J, Fan HQ, Unnerstall U, et al. (2004) Transcriptional control in the segmentation gene network of *Drosophila*. PLoS Biology 2: e271.

15. Pankratz MJ, Busch M, Hoch M, Seifert E, Jäckle H (1992) Spatial control of the gap gene *knirps* in the *Drosophila* embryo by posterior morphogen system. Science 255: 986-989.

16. Riddihough G, Ish-Horowicz D (1991) Individual stripe regulatory elements in the *Drosophila hairy* promoter respond to maternal, gap, and pair-rule genes. Genes and Development 5: 840-854.

17. Langeland JA, Carroll SB (1993) Conservation of regulatory elements controlling *hairy* pair-rule stripe formation. Development 117: 585-596.

18. Häder T, Rosée AL, Ziebold U, Busch M, Taubert H, et al. (1998) Activation of posterior pair-rule stripe expression in response to maternal *caudal* and zygotic *knirps* activities. Mechanisms of Development 71: 177-186.
