# Supplementary material for: Rearrangements of 2.5 Kilobases of Noncoding DNA from the Drosophila even-skipped Locus Define Predictive Rules of Genomic cis-Regulatory Logic
Source: PLoS Genet. 2013 Feb 28;9(2):e1003243. doi: 10.1371/journal.pgen.1003243 (PMC3585115; doi:10.1371/journal.pgen.1003243)
Supplement: Table S5 — Drosophila and Sepsid Species Abbreviations. For each full species name, the first word indicates the genus and the second word indicates species. (PDF) [file pgen.1003243.s012.pdf]

Table S5. *Drosophila* and Sepsid Species Abbreviations

Abbreviation	Species	Figure index
yak	*Drosophila yakuba*	(4C1)
pse	*Drosophila pseudoobscura*	(4C2)
ere	*Drosophila erecta*	(4C3)
ore	*Drosophila orena*	(4C4)
tei	*Drosophila teissieri*	(4C5)
tak	*Drosophila takahashi*	(4C6)
mau	*Drosophila mauritiana*	(4C7)
sec	*Drosophila sechellia*	(4C8)
per	*Drosophila persimilis*	(4C9)
sim	*Drosophila simulans*	(4C10)
ana	*Drosophila ananassae*	(4C11)
vir	*Drosophila virilis*	(4C12)
pic	*Drosophila picticornis*	(4C13)
gri	*Drosophila grimshawi*	(4C14)
moj	*Drosophila mojavensis*	(4C15)
wil	*Drosophila willistoni*	(4C16)
cyn	*Sepsis cynipsea*	(4E1,7)
put	*Themira putris*	(4E2,8)
sup	*Themira superba*	(4E3,9)
dsp	*Dicranosepsis sp.*	(4E4,10)
min	*Themira minor*	(4E5,11)
pun	*Sepsis punctum*	(4E6,12)

For each full species name, the first word indicates the genus and the second word indicates species.
